# The New Frontier of Quality Evaluation for Visual Sensors: A Survey of Large Multimodal Model-Based Methods

**DOI:** 10.3390/s26082530

**Published:** 2026-04-20

**Authors:** Qihang Ge, Xiongkuo Min, Sijing Wu, Yunhao Li, Guangtao Zhai

**Affiliations:** School of Integrated Circuits, Shanghai Jiao Tong University, Shanghai 200240, China; qihang.ge@sjtu.edu.cn (Q.G.);

**Keywords:** visual quality assessment, AI-generated content, large multimodal models

## Abstract

Visual quality assessment is entering a new frontier as media evolve from static images to temporally dynamic videos and 3D content. These visual signals are typically captured by sensing devices such as cameras and depth sensors, whose acquisition characteristics significantly influence perceptual quality. Traditional quality models, including distortion-centric and regression-based approaches, perform well on conventional degradations but struggle to evaluate higher-level attributes such as semantic plausibility and structural coherence in modern AI-generated and multimodal scenarios. The emergence of large multimodal models (LMMs), including vision–language models (VLMs) and multimodal large language models (MLLMs), reshapes the evaluation paradigm by enabling semantic grounding, instruction-driven assessment, and explainable reasoning. This survey presents a unified perspective on visual quality assessment for sensor-captured visual data across image, video, and 3D modalities. We review conventional deep learning approaches and recent LMM-based methods, highlighting how multimodal fusion and language-conditioned reasoning transform quality assessment from scalar prediction to perceptual intelligence. Finally, we discuss key challenges and future opportunities for building efficient, robust, and sensor-aware visual quality assessment systems.

## 1. Introduction

Visual quality assessment is critically important in modern multimedia and communication systems, driven by the increasing demand for high-quality visual content and enhanced quality of experience (QoE) [1]. Objective quality evaluation plays a central role in applications such as compression, restoration, transmission, content generation, and 3D reconstruction, where perceptual fidelity serves as a fundamental benchmark for algorithm design and system optimization.

In many practical scenarios, visual data originate from sensing devices such as mobile cameras, surveillance cameras, autonomous driving sensors, and RGB-D imaging systems. Imperfections in these sensing pipelines—including sensor noise, exposure instability, motion blur, and optical distortions—can significantly affect the perceptual quality of captured visual signals [2,3].

Traditionally, visual quality assessment has been divided into subjective and objective paradigms [4,5]. Subjective assessment collects human perceptual opinions under standardized viewing conditions and provides reliable ground-truth labels. However, its high cost and limited scalability restrict practical deployment. To enable automated and efficient evaluation, objective models have been developed to approximate perceptual judgments computationally. Early approaches relied on handcrafted perceptual priors, natural scene statistics (NSS), and statistical modeling [6,7,8,9,10,11,12], while subsequent deep learning methods shifted toward data-driven representation learning, distribution learning, and supervised regression [13,14,15,16,17,18]. These conventional approaches have substantially improved prediction accuracy, yet they remain largely distortion-centric and scalar-regression-oriented.

Over time, the scope of visual quality assessment has expanded from images to videos and 3D content [19,20,21,22,23,24]. Image quality assessment (IQA) operates under fixed spatial observation, focusing on structural fidelity and perceptual naturalness within a single frame. Video quality assessment (VQA) introduces temporal integration, where motion consistency, temporal masking, and perceptual memory jointly shape quality judgments [19,20,21,25,26]. 3D quality assessment (3DQA) further complicates the problem through viewpoint-conditioned and rendering-mediated perception: visual quality is not observed from a single static view, but depends on viewpoint selection, visibility, shading, and, in dynamic scenarios, temporal coherence [22,23,24,27]. As visual content becomes increasingly diverse and high-dimensional, distortion-centric modeling alone becomes insufficient for capturing complex semantic and contextual factors, particularly in AI-generated content (AIGC) [28,29,30,31,32,33,34,35].

The rise of large multimodal models (LMMs) has opened a new frontier for visual quality assessment [36,37,38,39,40,41]. In this paper, we use LMMs as an umbrella term encompassing both vision–language models (VLMs) and multimodal large language models (MLLMs).

VLMs, typically represented by models such as CLIP [36] and Flamingo [37], aim to bridge visual content and textual descriptions through cross-modal modeling. In quality assessment, they enable prompt-based evaluation by mapping images or videos to quality-related semantic concepts [42,43,44,45,46,47,48]. However, such approaches largely remain within a similarity-based or regression-oriented paradigm. In contrast, MLLMs extend this capability by incorporating large language models (LLMs) as reasoning backbones, enabling instruction-following, contextual understanding, and multi-step inference. This allows quality assessment to move beyond static score prediction toward prompt-conditioned evaluation, comparative reasoning, explanation generation, and quality-aware critique [49,50,51,52,53,54,55,56,57,58,59,60].

**Figure 1:**
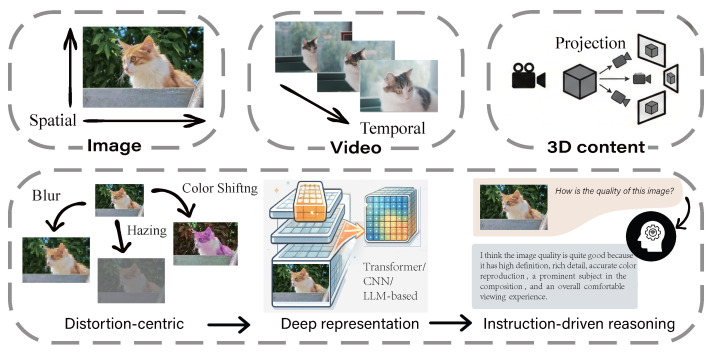
Overview of visual quality assessment across modalities and the paradigm shift enabled by LMMs. The upper row illustrates three major modalities of sensor-captured visual data: images (spatial), videos (spatio-temporal), and 3D content (intrinsic and projection-based perception). The lower row depicts the evolution of quality assessment paradigms, progressing from distortion-centric modeling (e.g., blur, noise, color shifting), to deep representation learning (e.g., CNNs and Transformers), and further to LMM-driven evaluation.

This evolution signals a broader transformation in visual evaluation—from distortion-sensitive regression toward semantically grounded and reasoning-capable assessment. Rather than relying solely on predefined distortion measures, modern approaches increasingly integrate perceptual cues with high-level semantic interpretation and language-conditioned criteria. As illustrated in Figure 1, visual quality assessment evolves along both modal complexity (from images to videos and 3D content) and methodological progression (from distortion-centric modeling to deep learning and further to LMM-driven reasoning). In particular, the paradigm shift is not only reflected in improved predictive performance, but also in a fundamental transition from scalar regression toward semantically grounded and instruction-driven evaluation [49,54,57,60].

In this survey, we provide a structured and comprehensive review of LMM-driven visual quality assessment across three major modalities: image, video, and 3D content. For each domain, we first introduce the task characteristics and dataset evolution, then summarize conventional modeling approaches, and finally examine recent advances enabled by LMMs. By organizing the literature along both modality and methodological progression, we aim to clarify the shared structural trends underlying visual evaluation and highlight emerging challenges in calibration, robustness, and scalability. To provide a more complete evaluation perspective, we further summarize commonly used evaluation metrics and benchmarking protocols in Section 4.6.

## 2. Image Quality Assessment: From Deep Learning to LMMs

### 2.1. IQA Task Description

IQA studies computational prediction of human judgments on the perceptual quality of an image [6,11,13]. Given an input image $I\in R^{H\times W\times C}$ acquired by visual sensing devices such as mobile cameras, surveillance systems, or other imaging platforms, IQA seeks to learn a mapping:

(1) $$f_{\theta }:I\to q,$$

where q∈R denotes a predicted perceptual quality score. In standard benchmark settings, *q* approximates the Mean Opinion Score (MOS) obtained from subjective experiments conducted under standardized protocols [4,5]. The learning objective is therefore commonly formulated as a regression problem:

(2) $$\min_{\theta }\;L\left(f_{\theta }\left(I\right),\mathrm{MOS}\right),$$

where θ denotes model parameters and L measures the discrepancy between the predicted score and human ratings. Although this formulation appears simple, the underlying perceptual factors are diverse in practice, spanning sensor noise, defocus blur, compression artifacts, exposure variation, ISP processing, enhancement artifacts, and domain-specific degradations introduced by different imaging conditions [61].

#### 2.1.1. Perceptual Characteristics of Image Quality

Unlike semantic recognition tasks, IQA is concerned not with object categories or scene labels but with how faithfully an image is perceived by human observers. Quality perception in static images exhibits several well-known properties. First, perceptual sensitivity is spatially non-uniform and multi-scale: distortions occurring in salient or structurally important regions may dominate the overall judgment. Second, the relationship between physical distortion strength and perceived degradation is highly nonlinear due to masking effects, contrast sensitivity, and contextual interactions [6]. Third, perceptual responses are content-dependent, meaning that the same distortion level may be judged differently depending on scene structure, texture statistics, and semantic layout [11]. These properties explain why effective IQA models typically need to capture both low-level fidelity cues and higher-level contextual modulation.

#### 2.1.2. Structural Scope and Limitations

The conventional IQA formulation primarily addresses spatial perceptual quality under fixed viewing conditions and static observation. In this setting, perception is modeled from a single image without temporal integration, viewpoint change, or explicit user interaction. This formulation has supported a mature body of work, including handcrafted methods, deep regression models, and a growing number of specialized IQA approaches for particular image domains [6,11,13,15,16]. At the same time, it also imposes a relatively narrow output form, typically a scalar score, which may be insufficient when quality criteria become scenario-dependent, semantically conditioned, or explanation-oriented [49,52,54]. This tension motivates the broader discussion in this survey: not because conventional IQA has been solved away but because emerging visual sensor scenarios increasingly call for quality assessment frameworks that are more flexible, interpretable, and adaptable. Table 1 summarizes representative image-quality datasets discussed in this section.

### 2.2. Evolution of IQA Datasets

The development of IQA methods has been closely intertwined with the evolution of benchmark datasets (Table 1). Early synthetic distortion datasets such as LIVE [61], TID2008 [62], and TID2013 [63] established controlled evaluation protocols with predefined distortion types, including blur, noise, and compression artifacts. These datasets reinforced a distortion-centric paradigm in which perceptual quality was largely attributed to measurable low-level impairments. KADID-10K [68] further expanded distortion diversity and benchmark scale, supporting data-driven regression under synthetic settings.

The release of large-scale authentic datasets marked a major transition. KonIQ-10k [2] introduced in-the-wild images containing complex mixtures of capture noise, compression artifacts, exposure problems, and device-dependent degradations. Such distortions often originate from heterogeneous acquisition pipelines, including smartphone cameras, consumer imaging devices, and unconstrained post-processing. Unlike synthetic benchmarks, these images exhibit compound and non-stationary artifacts that cannot be cleanly categorized, thereby pushing IQA toward more generalizable perceptual representation learning.

More recently, generative and enhancement-oriented datasets have further expanded the scope of IQA. General AIGC benchmarks such as AGIQA-3K [28], AIGIQA-20K [29], and PKU-AIGIQA-4K [74] extend quality annotation from low-level fidelity toward realism, semantic plausibility, and prompt faithfulness. Beyond general-purpose generation, AGHI-QA [76] highlights a human-centric setting in which body structure, local identity cues, and semantic consistency jointly shape perceived quality, while AIGI-VC [75] reflects the growing interest in communication-oriented perception of generated imagery. Together, these datasets show that modern IQA supervision increasingly couples perceptual fidelity with alignment- and realism-related criteria. Accordingly, modern IQA datasets no longer evaluate distortion magnitude alone but increasingly define quality in relation to realism, alignment, and contextual coherence. This broader benchmark design naturally motivates models that combine distortion sensitivity with higher-level perceptual and semantic understanding.

### 2.3. Conventional Modeling Approaches for IQA

Before the emergence of LMM-based paradigms, IQA was primarily developed through handcrafted perceptual modeling and deep learning-based regression frameworks. These approaches aim to approximate human perceptual responses either by explicitly modeling distortion statistics and structural fidelity or by learning quality-aware representations from annotated datasets. Although differing in methodology, they share a common characteristic: quality prediction is formulated as a regression problem toward subjective scores without explicit semantic reasoning or language-grounded interaction.

#### 2.3.1. Handcrafted IQA Methods

Early IQA research focused on designing perceptually motivated quality metrics grounded in signal processing and NSS. Full-reference (FR) methods compare a distorted image with its pristine reference to quantify fidelity loss. Representative indices include PSNR (pixel-wise error), SSIM and MS-SSIM (structural similarity across scales) [6,7], VIF [8] (information fidelity), FSIM [9] (feature similarity), and GMSD [10] (gradient magnitude similarity deviation). These metrics emphasize structural consistency, contrast sensitivity, and multi-scale perception, and remain widely used as evaluation baselines.

For no-reference (NR) scenarios, NSS-based approaches model deviations from statistical regularities observed in natural images. Methods such as BRISQUE [11] and NIQE [12] extract handcrafted features from spatial-domain statistics and employ simple regressors to estimate quality. By capturing distortion-induced distribution shifts, these approaches achieve reasonable generalization without reference images.

While these methods provide interpretability and computational efficiency, their reliance on predefined distortion models or handcrafted statistics limits adaptability to complex, mixed, or semantically driven degradations.

#### 2.3.2. Conventional Deep Learning-Based IQA Methods

The emergence of deep learning marked a turning point in IQA by shifting from handcrafted feature engineering to data-driven representation learning. Early works such as CNNIQA [13] demonstrated that convolutional neural networks can directly regress perceptual quality scores from image patches without explicitly modeling distortion statistics. This end-to-end paradigm established deep regression as a dominant framework for blind IQA.

Shortly afterward, NIMA [14] reformulated quality prediction as a rating-distribution learning problem. Instead of regressing a single MOS value, NIMA predicts a discrete probability distribution over ordered quality levels using a softmax output and optimizes an Earth Mover’s Distance-based loss to preserve ordinal structure. The MOS can then be computed as the expectation of the predicted distribution, providing a probabilistic interpretation of perceptual variability.

To improve cross-distortion robustness, DBCNN [15] introduced bilinear pooling to fuse content-aware and distortion-aware features, enhancing representation capacity across synthetic distortion types. HyperIQA [16] further incorporated a hyper-network that dynamically generates image-adaptive regression parameters, improving flexibility under authentic distortions. In specialized settings, face-centric quality studies such as FaceQnet [77] and SER-FIQ [78] further show that task-aware quality representations can complement generic natural-image IQA priors. UNIQUE [17] explored uncertainty-aware quality estimation by leveraging unsupervised feature learning and confidence modeling, improving robustness in both laboratory-controlled and in-the-wild settings. More recent efforts further strengthen authentic distortion generalization. StairIQA [18] addressed cross-database generalization by combining hierarchical feature fusion with iterative mixed-dataset training, while representative authentic datasets such as KonIQ-10k [2] and SPAQ [79] continue to motivate models that remain stable under diverse real-world degradations.

Representative RGB-D methods and domain-specific IQA models consistently suggest that image quality modeling should go beyond generic natural-image priors and explicitly account for modality reliability, imaging physics, and application-specific degradations. In RGB-D settings, Chen et al. [80] introduced a depth contribution assessment subnet to identify depth regions that provide beneficial complementary cues for RGB-D fusion, thereby suppressing low-quality or misleading depth responses. Ji et al. [81] further proposed a depth calibration and fusion framework, which first calibrates noisy raw depth maps and then performs cross-reference fusion with RGB features. These studies indicate that multimodal image quality modeling in RGB-D scenarios depends critically on depth reliability estimation and adaptive cross-modal fusion. Similar observations can also be found in other specialized image domains. For tone-mapped images, Yeganeh and Wang [82] proposed the classical TMQI, which combines structural fidelity and statistical naturalness for objective quality assessment, while Yang et al. [83] further introduced a blind quality assessment framework based on multi-exposure sequences. For compressed images, Liu et al. [84] proposed SwinIQA to learn a Swin-Transformer-based perceptual distance for compressed IQA. In underwater imaging, Yang and Sowmya [85] proposed UCIQE, a representative NR metric for evaluating underwater-specific color distortion, contrast attenuation, and visibility degradation, while Xian et al. [86] proposed PIGUIQA, which integrates physical imaging modeling with perceptual learning to better characterize attenuation and backward scattering. For sonar images, Zhang et al. [87] proposed a deep neural network for sonar image quality evaluation, while Chen et al. [88] introduced a blind contour-aware quality model to capture sonar-specific texture, structure, and target interpretability. Collectively, these studies suggest that once IQA moves beyond generic natural-image settings, effective quality modeling often requires sensor-aware or domain-aware designs rather than uniformly applying general-purpose quality criteria. Table 2 provides a concise comparison of representative IQA approaches.

Overall, deep learning substantially enhances representation capacity and generalization compared with handcrafted approaches. However, these models remain primarily supervision-driven and regression-centric, with limited semantic grounding or interactive controllability limitations that motivate the LMM-based paradigms discussed in Section 2.4.

### 2.4. Large Multimodal Model Approaches for IQA

VLMs and MLLMs fundamentally reshape IQA by introducing cross-modal alignment, semantic grounding, and instruction-following capabilities. Following the distinction between VLMs and MLLMs introduced in Section 1, we categorize LMM-based IQA methods into alignment-based and reasoning-based paradigms.

#### 2.4.1. VLM-Based IQA Methods

The earliest LMM-based IQA approaches adapt pretrained VLMs, particularly CLIP [36], to become quality-aware through prompt engineering and lightweight finetuning.

CLIP-IQA [42] demonstrated that carefully designed quality-related textual prompts can elicit perceptual “look-and-feel” priors from CLIP in a zero-shot manner. By measuring similarity between image embeddings and quality-descriptive text embeddings, CLIP-IQA showed that VLMs implicitly encode perceptual cues without MOS supervision. Building upon this idea, QA-CLIP [43] introduced learnable prompt tokens and staged finetuning to better align image–text embeddings with fine-grained quality levels. Rather than relying purely on handcrafted prompts, QA-CLIP optimized prompt representations and regression heads to enhance discriminability across quality strata. To reduce dependence on subjective labels, QualiCLIP [89] proposed self-supervised quality-aware alignment by enforcing monotonic ranking constraints under controlled degradations. This formulation leverages synthetic distortion hierarchies to guide CLIP finetuning without explicit MOS labels, improving scalability. Beyond scalar alignment, LIQE [44] formulated IQA as a probabilistic multi-task problem incorporating semantic and distortion-related objectives. By jointly modeling quality and auxiliary perceptual attributes within a vision–language framework, LIQE improved cross-database robustness while maintaining efficiency. ZEN-IQA [45] performs zero-shot NR-IQA with explainable outputs by leveraging VLMs and carefully designed quality prompts to infer overall and attribute-level degradation cues without IQA-specific training.

Overall, VLM-based approaches improve visual–semantic alignment but largely remain within regression-style prediction frameworks.

#### 2.4.2. MLLM-Based IQA Methods

More recent work leverages MLLMs to reformulate IQA as an instruction-following and reasoning-oriented task. Instead of treating IQA as a fixed scalar-regression task, MLLM-based approaches enable prompt-conditioned evaluation and, in many cases, explainable inference:

(3) $$\left(\hat{q},r\right)=M_{\Theta }\left(I,p\right),$$

where *p* specifies evaluation criteria and *r* denotes optional rationale or attribute-level feedback.

PromptIQA [49] conditions models on image-score demonstrations, enabling quality assessment that adapts outputs to given textual requirements. Comparative reasoning provides a natural interface for MLLMs. 2AFC prompting [50] queries an MLLM with image pairs and aggregates pairwise preferences via probabilistic ranking models. Compare2Score [51] further trains MLLMs to produce structured relative judgments and converts preference matrices into continuous quality scores, bridging comparative reasoning with MOS regression. Benchmark studies such as Q-Bench [55] and VisualCritic [56] also reveal that general-purpose MLLMs still exhibit nontrivial gaps in subtle quality perception, thereby motivating task-specific instruction tuning and grounded reasoning. Reinforcement learning-based quality reasoning has recently emerged as another line of development. Q-Insight [93] introduces a visual reinforcement learning framework for image quality understanding, jointly optimizing score regression and degradation perception to improve both quality prediction and interpretability. VisualQuality-R1 [94] further reformulates NR-IQA as a reasoning-induced ranking problem, leveraging reinforcement learning to model relative quality judgments and generate human-aligned quality descriptions. These methods extend MLLM-based IQA beyond prompt-conditioned scoring toward more explicit reasoning-oriented quality modeling.

Instruction tuning strengthens controllable perceptual understanding. Q-Instruct [53] constructs low-level visual instruction datasets to improve distortion sensitivity in MLLMs. DepictQA [52] reframes IQA from predicting a single scalar score to generating structured quality descriptions and comparative rationales, improving interpretability and diagnostic usefulness. DepictQA-Wild [92] extends descriptive IQA to in-the-wild settings with greater content diversity, while DeQA-Score [91] incorporates distribution-based regression to bridge explanation generation with quantitative scoring. Grounding-IQA [54] introduces region-aware quality reasoning by aligning global judgments with spatially grounded visual evidence. It enhances fine-grained interpretability through localized distortion attribution. iDETEX [90] further unifies scoring with grounded localization and detailed critique generation, aligning quality prediction with spatial attribution.

In addition to these advances, quality assessment methods tailored to AI-generated images have also attracted increasing attention in recent years. Specifically, Li et al. [30] proposed ELIQ, a label-free framework that leverages intrinsic distributional and evolutionary consistency cues to assess the quality of AI-generated images without requiring subjective annotations. Complementarily, PKU-AIGIQA-4K [74] and the NTIRE 2025 challenge [31] further push standardized evaluation for AI-generated image quality and text–image alignment.

To facilitate a concise comparison between representative conventional and LMM-based IQA methods, Table 3 summarizes their performance on widely used synthetic and authentic distortion datasets. The comparison shows that standard in-domain IQA is already a highly mature problem: several conventional deep models achieve very strong agreement with human opinion scores, and recent LMM-based methods, while competitive, do not yet exhibit uniformly dominant advantages over the best conventional baselines across all datasets. Therefore, the benchmark evidence in this section is better interpreted as showing an expansion of the methodological landscape of IQA, rather than a simple replacement of existing approaches by LMMs.

### 2.5. Summary of IQA Progression

Overall, the development of IQA reflects a gradual expansion from distortion-centric prediction toward richer forms of perceptual modeling. Early handcrafted methods focused on explicit signal fidelity and NSS, while conventional deep models substantially improved representation learning and robustness on both synthetic and authentic distortion benchmarks. As shown by the benchmark comparison above, this line of research is already highly mature, with several non-LMM methods achieving very strong agreement with human opinion scores.

Recent VLM- and MLLM-based approaches further extend IQA beyond scalar score prediction by introducing promptable and semantically aware quality assessment. In this sense, they complement rather than replace existing IQA paradigms.

## 3. Video Quality Assessment: From Deep Learning to LMMs

### 3.1. VQA Task Description

VQA, while conceptually rooted in IQA, is not merely image quality prediction applied frame by frame. Its defining challenge is that perceived quality is formed over time: motion, temporal persistence, and evolving distortions jointly shape the final judgment.

Given a video sequence $V={\left\{I_{t}\right\}}_{t=1}^{T}$, captured by devices such as mobile cameras, surveillance systems, and vehicle-mounted sensors, VQA aims to learn a mapping:

(4) $$f_{\theta }:V\to q,$$

where *q* approximates subjective quality labels obtained from laboratory studies or crowdsourcing protocols. A straightforward extension of IQA applies frame-level predictors to sampled frames and averages the outputs. However, such frame-wise strategies neglect inter-frame dependencies and cannot faithfully represent how human observers accumulate quality impressions over time. Effective VQA must therefore model not only spatial fidelity within individual frames but also the temporal organization of perceptual evidence across frames.

#### 3.1.1. Perceptual Characteristics of Video Quality

Compared with static IQA, VQA is distinguished by a temporally formed perceptual target rather than a temporally extended input alone.

First, perceptual quality is accumulated over time rather than determined instantaneously. Observers do not judge a video as an unordered collection of frames; instead, local impressions are integrated into a global quality judgment through temporal pooling and perceptual memory. As a result, brief but salient degradations may disproportionately influence the final opinion, and quality prediction cannot be reduced to simple frame averaging [20,21].

Second, artifact visibility is conditioned by motion. Motion may partially mask some spatial impairments, yet it can also amplify distortions that are weak in still images but highly noticeable in dynamic viewing, such as flicker, judder, ghosting, and temporal aliasing. Consequently, VQA must account for motion-conditioned sensitivity and temporal coherence, rather than treating distortion magnitude as a purely spatial quantity [19,25].

Third, authentic videos often exhibit mixed and non-stationary degradations introduced by capture, processing, compression, and transmission. Sensor noise, motion blur, shakiness, focus instability, exposure fluctuation, and bitrate variation may co-occur and evolve over time, interacting with scene dynamics in a content-dependent manner. This makes the quality signal temporally heterogeneous and often more ambiguous than the relatively stationary settings considered in conventional IQA [20,21,26].

#### 3.1.2. Structural Scope and Modeling Implications

The historical development of VQA reflects a shift from static fidelity estimation to temporally organized perceptual inference. Early VQA methods had already shown that motion-aware analysis is essential: quality must be evaluated not only in space but also along motion trajectories and through spatio-temporal statistics [19,25]. With the rise of user-generated content (UGC), the field further moved toward models that address authentic capture distortions, long-range temporal dependencies, and efficient temporal sampling, as represented by methods such as TLVQM, VSFA, and FAST-VQA [20,21,26].

From the perspective of this survey, the core significance of VQA is therefore not simply that videos are longer than images, but that their perceptual quality is formed through temporal interaction, persistence, and variation. This temporal formation mechanism fundamentally distinguishes VQA from IQA and explains why subsequent advances in datasets, architectures, and evaluation protocols increasingly emphasize temporal reasoning rather than static distortion estimation alone.

At the same time, most conventional VQA methods still ultimately compress the viewing process into a single scalar score. Such a formulation is effective for many benchmark settings, yet it becomes increasingly strained when perceptual quality is entangled with semantic continuity, generated motion plausibility, or richer explanation demands. This limitation motivates the LMM-based VQA paradigms discussed in Section 3.4.

### 3.2. Evolution of VQA Datasets

The evolution of VQA datasets reflects a clear expansion from controlled codec distortions to authentic temporal experience and, more recently, semantically complex generated videos (Table 4).

Early benchmarks such as LIVE-VQA [95] and CSIQ-VQA [112] mainly focus on compression and packet-loss impairments under relatively controlled conditions. These datasets encouraged distortion-aware modeling with relatively simple temporal pooling strategies, where video quality was largely treated as a temporally extended version of image fidelity assessment.

The introduction of UGC-oriented datasets marked a major shift. KoNViD-1k [98], LIVE-VQC [99], and YouTube-UGC [100] expose mixed authentic degradations caused by diverse capture devices, motion patterns, scene contents, and editing pipelines. These datasets demonstrate that real-world video quality is shaped by temporally varying and content-dependent distortions rather than by isolated codec artifacts alone. Large-scale datasets such as LSVQ [101] further support data-driven temporal representation learning and more reliable cross-content evaluation. Large-scale and in-the-wild studies also continue to broaden the diversity of video sources and temporal distortions encountered in practice.

A related branch of dataset development emphasizes streaming QoE. Datasets such as LIVE-NFLX [102] incorporate bitrate adaptation, delivery dynamics, and playback continuity, showing that perceptual quality depends not only on spatial fidelity within frames but also on temporal stability, interruption patterns, and viewing continuity across time. This branch broadens VQA from artifact recognition toward user-centered modeling of temporal viewing experience.

More recently, AI-generated benchmarks have pushed VQA toward semantically richer and temporally more challenging scenarios. Broad evaluation suites such as FETV [32], VBench [33], EvalCrafter [34], TC-Bench [109], DEVIL [110] increasingly emphasize temporal compositionality, motion plausibility, semantic consistency, and holistic generation quality. HVEval [35] further specializes this trend in human-centric videos, where local facial realism, identity consistency, and articulated motion strongly influence perception. In these benchmarks, video quality is increasingly entangled with semantic continuity, motion plausibility, and generation faithfulness.

Overall, the evolution of VQA datasets shows that VQA is not merely an image-based problem with more frames. Instead, benchmark design has progressively expanded from controlled distortion evaluation to authentic temporal perception, streaming experience, and AI-generated video realism. This broader dataset landscape explains why effective VQA models must increasingly address temporal organization, motion-conditioned perception, and semantic video consistency rather than relying on frame-level distortion analysis alone.

### 3.3. Conventional Modeling Approaches for VQA

Conventional modeling VQA approaches can be broadly categorized into classical handcrafted methods and deep learning-based representation learning frameworks.

#### 3.3.1. Handcrafted VQA Methods

Early VQA methods explicitly modeled spatio-temporal statistics and motion-aware perceptual cues. MT-STQA [25] is a FR-VQA metric that models human-visual sensitivity to motion by jointly analyzing spatial distortion and temporal motion information. Reduced-reference (RR) designs such as STRRED [113] transmitted compact statistical features and computed entropic differences to estimate quality degradation. For NR settings, Video BLIINDS [19] leveraged spatio-temporal NSS and motion coherence features to balance efficiency and prediction accuracy. VIIDEO [114] models intrinsic statistical regularities in natural videos without using opinion labels, while STEM [115] combines a temporal straightness-based perceptual measure with blind spatial quality cues to improve generalization on authentic UGC videos.

These classical approaches explicitly encode perceptual priors but rely on predefined distortion statistics and limited adaptability to complex in-the-wild degradations.

#### 3.3.2. Conventional Deep Learning-Based VQA Methods

Deep learning shifted VQA toward representation-driven modeling of temporal dynamics. VSFA [21] employed CNN feature extraction followed by GRU-based temporal modeling to capture long-term dependencies and perceptual memory effects, establishing a recurrent-learning paradigm for UGC videos. Subsequent works explored stronger spatio-temporal architectures. FAST-VQA [26] introduced fragment-based sampling and attention-based aggregation to reduce computational cost while preserving temporal cues. Complementary studies such as RAPIQUE [116] further emphasized efficient content-aware feature reuse and quality-aware pretraining for in-the-wild videos. DisCoVQA [117] modeled distortion–content interaction through Transformer-based temporal coupling. STI-VQA [118] further emphasized spatial-motion interaction by tokenizing distortion statistics and motion features within a Transformer framework. SAMA [119] further proposed a scaling-and-masking sampling strategy for image and video quality assessment, aiming to preserve both local details and global semantics within a regular input size. Table 5 compares representative VQA approaches across classical, deep, and LMM-based paradigms.

To address perceptual complexity beyond a single MOS score, DOVER [120] disentangles technical distortion perception from aesthetic preference by modeling quality from both technical and aesthetic perspectives. Building on the need for interpretability, FineVQ [104] introduces fine-grained, attribute-level supervision, incorporating multi-dimensional quality ratings (e.g., color, noise, blur, temporal) and degradation-type annotations (e.g., over-exposure, blur, jitter, frame drop, stall) to enable quality attribution and more explainable assessment. To address practical variability in video formats, ModularBVQA [121] incorporates resolution-aware spatial rectifiers and frame-rate-aware temporal rectifiers to explicitly compensate for differences in spatial and temporal sampling conditions. Controlled analyses of spatio-temporal modeling and in-the-wild video quality datasets further investigated dataset bias and capacity limits in deep VQA architectures [21,125].

Overall, deep learning substantially improves spatio-temporal representation capacity compared with handcrafted approaches. However, these methods remain largely regression-centric and supervision-dependent, motivating the transition toward LMM-based reasoning and instruction-driven evaluation discussed in Section 3.4.

### 3.4. Large Multimodal Model Approaches for VQA

Instead of treating VQA as a fixed scalar-regression task, LMM-based approaches allow quality criteria to be specified in natural language and, in some cases, generate structured explanations alongside scores. These paradigms are particularly valuable for AI-generated and human-centric videos, where degradations may be semantic (implausible content) or temporal (identity drift, motion discontinuity), and cannot be exhaustively enumerated as distortion labels.

#### 3.4.1. VLM-Based VQA Methods

VLM-based VQA methods adapt pretrained vision–language encoders to inject semantic awareness into quality prediction without heavy MOS supervision.

BUONAVISTA [46] introduced an opinion-unaware VQA framework that measures semantic affinity between video embeddings and quality-related textual prompts. By combining prompt-derived semantic scores with conventional technical indices, BUONAVISTA demonstrated that CLIP-like encoders encode perceptual plausibility cues useful for quality estimation. BVQI [47] further refined text-prompted semantic criteria by designing more robust prompt formulations and introducing localized semantic affinity measures. These mechanisms improve sensitivity to high-level perceptual failures beyond low-level distortion statistics, particularly in AI-generated or semantically complex videos. More recently, CAMP-VQA [126] extended this line of research from prompt-based semantic matching to caption-guided multimodal regression. It uses quality-aware prompts to guide a pretrained BLIP-2 model to generate fine-grained quality captions, and then fuses semantic, temporal, and spatial features through dedicated branches before regressing final MOS scores. Compared with earlier CLIP-affinity methods, CAMP-VQA preserves the regression-style output paradigm while offering stronger artifact-semantic awareness and better modeling of compressed UGC videos.

Such VLM-based approaches act as a transitional bridge between conventional deep VQA and fully instruction-driven MLLM evaluators. They enhance semantic sensitivity while largely preserving regression-style score outputs.

#### 3.4.2. MLLM-Based VQA Methods

More recent approaches leverage MLLMs to reformulate VQA as an instruction-following and reasoning task.

LMM-VQA [57] casts MOS prediction into a VQA-style question-answering problem, aligning spatio-temporal visual tokens with a MLLM to output quality levels conditioned on textual prompts. This design allows flexible quality specification and moves beyond fixed regression heads. CP-LLM [58] introduced dual vision encoders (context-level and pixel-level) and multi-task objectives spanning scoring, description generation, and pairwise comparison. By integrating contextual and fine-grained cues, CP-LLM improves sensitivity to subtle distortions while maintaining semantic reasoning capability. VQAThinker [122] introduces reinforcement learning with quality-aware reward designs to jointly optimize interpretable distortion analysis and robust score prediction. Pretraining strategies further enhance quality priors. Such pretraining aims to endow models with stable perceptual priors before task-specific adaptation.

MLLM-based VQA also expands evaluation targets beyond scalar scores. ${\mathrm{VQA}}^{2}$ [123] constructed dedicated instruction datasets for video quality perception, training models that interleave visual and motion tokens to support both scalar scoring and quality-related question answering. This work highlights the role of curated instruction data in strengthening controllable quality understanding. VQ-Insight [127] further explores reasoning-oriented quality understanding for AI-generated videos through progressive visual reinforcement learning, emphasizing multi-dimensional scoring and temporal modeling in AIGC-specific settings.

In human-centric and face-focused scenarios, FVQ [124] focuses on face-centric quality modeling, emphasizing identity preservation, facial detail fidelity, and temporal coherence across frames, thereby improving sensitivity to subtle distortions that affect facial authenticity and recognition consistency.

Table 6 summarizes the performance of representative conventional and LMM-based NR-VQA methods on five common UGC benchmarks. Compared with the corresponding IQA results, a different trend can be observed here: LMM-based methods already show clearer competitiveness, and in several cases, stronger overall performance, on common in-domain VQA benchmarks. In particular, native VQA-oriented MLLM methods such as LMM-VQA [57], ${\mathrm{VQA}}^{2}$ [123], and VQAThinker [122] achieve highly competitive, and in some cases, leading results across multiple datasets.

This difference suggests that the role of LMMs may be more substantial in VQA than in IQA. Unlike static image quality prediction, VQA requires the joint modeling of spatial distortion, temporal coherence, motion continuity, and semantic plausibility over time, making the task inherently more aligned with temporally contextualized and reasoning-aware architectures. At the same time, the table also indicates that these gains are not automatic: strong conventional methods such as FAST-VQA [26], DOVER [120], and SAMA [119] remain highly competitive, while transferred reasoning-oriented IQA models such as Q-Insight [93] and VisualQuality-R1 [94] fall clearly behind dedicated VQA designs. Therefore, the current evidence does not point to a universal replacement of conventional VQA pipelines, but it does suggest that LMM-based approaches have already become a particularly promising direction for VQA.

### 3.5. Summary of VQA Progression

In summary, the development of VQA reflects a transition from handcrafted spatio-temporal descriptors and shallow perceptual priors to increasingly expressive deep architectures and, more recently, LMMs. Early NR methods mainly relied on natural video statistics, motion coherence, and hand-crafted temporal features, while subsequent deep models substantially improved performance by learning richer spatial–temporal representations from large-scale UGC datasets.

As shown by the benchmark comparison above, recent LMM-based methods already exhibit a clearer advantage trend in VQA than what is currently observed in IQA. In particular, native VQA-oriented MLLM frameworks achieve highly competitive, and in some cases, leading performance across multiple common UGC benchmarks, suggesting that VQA may benefit more directly from architectures with stronger temporal context modeling and higher-level reasoning capacity. At the same time, strong conventional methods remain highly competitive, and the table also shows that not every reasoning-based quality model transfers equally well to VQA.

Therefore, the progression reviewed in this section is best understood as an evolution toward richer temporal, semantic, and reasoning-aware quality modeling. Rather than indicating a simple replacement of conventional VQA methods, current evidence suggests that LMMs have become a particularly promising direction for NR-VQA, especially when quality judgment depends on the joint understanding of spatial distortion, motion dynamics, and temporal consistency.

## 4. 3D Quality Assessment: From Deep Learning to LMMs

### 4.1. Task Description of 3DQA

3DQA concerns the perceptual evaluation of three-dimensional visual content, including point clouds, textured meshes, and volumetric reconstructions acquired by sensing systems such as LiDAR, RGB-D cameras, structured-light devices, and multi-view capture pipelines. Given a 3D asset *X*, 3DQA aims to learn a mapping:

(5) $$f_{\theta }:X\to q,$$

where q∈R approximates subjective quality scores obtained under human viewing protocols.

Unlike IQA and VQA, the perceptual target in 3D is defined over native geometric and attribute representations whose visual consequences are not trivially readable from Euclidean grids or temporal frames. The central challenge of 3DQA is therefore to assess how distortions in a 3D representation translate into perceptual quality degradation under human observation. Such perceptual evidence may be exposed through rendered views, viewpoint changes, and visibility conditions, but it may also be approximated directly from intrinsic geometric and attribute structures. In this sense, projection is an important perceptual interface rather than the sole defining basis of 3DQA.

#### 4.1.1. Perceptual Characteristics of 3D Quality

Compared with IQA and VQA, the distinctive challenge of 3DQA lies in the gap between native 3D representations and their perceptual manifestation. A 3D object is not observed as a single fixed signal; instead, its perceived quality depends on how structural and appearance distortions become visible, salient, and interpretable under incomplete observation.

First, 3D representations exhibit structural irregularity. Point clouds are unordered and often non-uniformly sampled, while meshes and reconstructed surfaces may contain missing regions, topological defects, or unstable local structures. The perceptual importance of these errors is highly uneven: distortions near contours, thin components, articulated parts, or high-curvature regions are often much more noticeable than those in visually redundant areas. Therefore, 3DQA must account not only for distortion magnitude but also for how structural errors are distributed over perceptually sensitive regions.

Second, geometry and appearance are tightly coupled in perception. Geometric perturbations can alter normals, silhouettes, shading, and occlusion relationships, thereby changing perceived appearance even when texture values remain unchanged. Conversely, color and texture degradations may amplify the visual salience of structural defects. Perceived quality is thus determined by the joint effect of geometric fidelity and attribute consistency rather than by either factor alone.

Third, 3D quality perception is observation-dependent. Because only part of a 3D object is visible under any given observation condition, factors such as viewpoint, visibility, occlusion, and rendering strategy affect how perceptual evidence is exposed. However, these factors should be understood as mechanisms for revealing quality-relevant evidence, rather than as the only valid representation space for assessment. Accordingly, practical 3DQA methods may reason about them explicitly through rendered views, or implicitly through native 3D representations that encode perceptually relevant structural cues.

#### 4.1.2. Structural Scope and Dynamic Extension

From this perspective, the canonical 3DQA problem is best understood as a perceptual assessment of native 3D distortions under observation-dependent evidence. Its central question is not simply how much a 3D representation deviates from a reference, nor solely how it appears under a particular rendering pipeline, but how geometric and attribute errors become perceptually meaningful to human observers. This broader formulation naturally supports multiple methodological routes, including intrinsic 3D modeling, projection-based perceptual modeling, and hybrid strategies that combine both.

Most existing 3DQA settings focus on static content, where the main challenge lies in reasoning over structural fidelity, geometry–appearance coupling, and incomplete perceptual exposure. Recent applications, however, increasingly involve dynamic 3D media such as dynamic point clouds, digital humans, and volumetric video. In these scenarios, temporal instability, flicker, topology inconsistency, and motion-dependent structural artifacts introduce an additional coherence dimension. Nevertheless, such temporal effects should be viewed as an extension of 3DQA to dynamic immersive content rather than the defining property of 3D perception itself.

Overall, the core significance of 3DQA is that perceptual quality must be inferred across a representation-to-perception gap: the object is stored in native 3D form, but its quality is judged through partially exposed perceptual consequences. This formulation explains why subsequent developments in datasets and models evolve along multiple directions, including rendered-view assessment, native 3D representation learning, and multimodal reasoning over geometry, appearance, and semantics. It also motivates the LMM-based 3DQA paradigms discussed in Section 4.4, where rendered observations, intrinsic 3D structure, and language-guided interpretation begin to be integrated within a unified framework.

### 4.2. Evolution of 3DQA Datasets

The evolution of 3DQA datasets (Table 7) reflects a gradual broadening of the task from controlled geometric distortion analysis to perceptual evaluation over richer 3D representations, observation conditions, and application scenarios.

Early point-cloud benchmarks such as G-PCD [130,131] and RG-PCD [132] mainly focus on geometric distortions under relatively controlled settings, enabling systematic analysis of spatial fidelity but offering limited coverage of attribute-related perception. Subsequent datasets incorporate color information, distortion diversity, and more realistic processing pipelines, including IRPC [134], SJTU-PCQA [23], WPC [135], and BASICS [137]. Large-scale NR settings such as LS-PCQA [3] further shift the emphasis toward robustness under realistic and mixed degradations.

Beyond static point clouds, the dataset landscape has expanded toward broader 3D content types and more diverse perceptual factors. Temporal 3DQA emerges with compressed or dynamic sequences, such as WPC2.0 [136] and DPCD [141], while volumetric video quality is represented by VsenseVVDB [133]. On the mesh side, CMDM [138] and textured mesh datasets such as TMQA [139] explicitly highlight geometry–texture interactions. These developments indicate that 3DQA is no longer confined to geometry-only fidelity estimation but increasingly concerns the joint perceptual effect of structure, attributes, and content type.

More recent datasets further extend 3DQA toward semantically sensitive and human-centric scenarios. Human-centric 3DQA becomes prominent in head and full-body digital human settings, such as SJTU-H3D [48], as well as dynamic and streaming scenarios, including DDHQA [140] and DHQA-4D [142]. In such settings, perceptual sensitivity is highly non-uniform, with semantically critical regions (e.g., face and articulated body parts) often dominating subjective judgments. Subjective evaluation may be conducted through rendered views or sequences, but the underlying assessment target remains the quality of native 3D content and its perceptual consequences.

Overall, the evolution of 3DQA datasets suggests that the field is moving from geometry-centric distortion evaluation toward representation-diverse, perception-oriented, and human-centric assessment. This broader dataset landscape naturally supports multiple methodological routes, including intrinsic 3D modeling, projection-based perceptual modeling, and hybrid approaches, and thereby motivates the progression from conventional deep models to LMM-driven 3DQA paradigms.

### 4.3. Conventional Modeling Approaches for 3DQA

Conventional 3DQA methods can be broadly grouped into two paradigms. Intrinsic 3D modeling operates directly on native geometry and attributes, aiming to characterize distortions in the original 3D representation. Projection-based perceptual modeling, by contrast, evaluates rendered observations that more closely mimic human viewing. Recent methods increasingly bridge the two by combining projection cues with auxiliary geometric representations.

#### 4.3.1. Intrinsic 3D Modeling

Intrinsic approaches assess quality directly in geometry–attribute space, without explicitly rendering the content into 2D observations. Early FR metrics mainly focus on robust geometric comparison under irregular sampling and non-uniform density. PCQM [22] combines point-to-surface distance estimation with quadric surface fitting and fuses geometry- and color-related components, thereby improving robustness to density variations while jointly measuring geometric and attribute degradation. MS-GraphSIM [143] further models local neighborhoods as multi-scale graphs and measures structural and color-gradient consistency across scales. Likewise, Point2Dist [144] compares local geometry and color neighborhoods through statistical point-to-distribution discrepancies, offering a more distribution-aware alternative to pointwise correspondence.

In the NR setting, intrinsic modeling has also been explored through learned quality representations directly extracted from distorted point clouds. LS-PCQA [3] not only introduced a large-scale point-cloud quality database but also proposed the ResSCNN backbone for learning quality-aware representations in native 3D space. Related work also investigated task-driven supervision, where VisionTasks-PCQA [145] uses the performance degradation of auxiliary vision tasks as a proxy signal for perceptual quality, thereby injecting machine-perception sensitivity into intrinsic quality modeling.

Overall, intrinsic methods are naturally suited to characterizing geometric perturbations, irregular sampling, and joint geometry–attribute distortions in the native 3D domain. However, because they do not explicitly model how the distorted content is actually observed after rendering, their connection to perceptual visibility is often indirect.

#### 4.3.2. Projection-Based and Hybrid Perceptual Modeling

Because human observers ultimately perceive 3D content through rendered appearances, projection-based modeling has become a dominant paradigm in both FR and NR 3DQA. SJTU-PCQA [23] established an influential 3D-to-2D projection framework by rendering point clouds into multiple views and aggregating projection-level quality evidence, demonstrating that rendered observations provide an effective perceptual interface for objective quality prediction. Deep NR models such as PQA-Net [24] continue this direction by extracting view-wise features from multi-view projections and learning a regression model for perceptual quality prediction.

Subsequent work improved this projection-based pipeline from different angles. IT-PCQA [146] transfers 2D IQA priors to point-cloud quality assessment through unsupervised domain adaptation on multi-perspective rendered views, revealing a practical bridge between image quality knowledge and 3D perceptual assessment. GMS-3DQA [147] addresses the efficiency bottleneck of multi-view modeling by introducing grid mini-patch sampling over multiple projections and aggregating the sampled content into a compact quality map for Transformer-based feature extraction. MovingCam-PCQA [148] further extends static multi-view observation into dynamic perceptual simulation by capturing point clouds as moving-camera videos and jointly modeling frame-level spatial evidence and clip-level temporal variation.

A more recent trend is to move from purely projection-based assessment toward hybrid perceptual modeling. MM-PCQA [27] explicitly combines point-cloud sub-model features with projected image features and fuses them through cross-modal attention, showing that native 3D structure and rendered appearance provide complementary quality evidence. CoPA [149] further improves NR point-cloud quality prediction through contrastive pretraining and semantic-guided multi-view fusion, highlighting the continued importance of data-efficient representation learning for 3D perceptual assessment. MPV-PCQA [150] extends this idea by jointly modeling point clouds and captured dynamic video, integrating intrinsic geometric information with dynamic rendered observations in a unified NR framework. Table 8 summarizes representative 3DQA approaches and their primary input modalities.

Beyond point clouds, related studies on other 3D content types also reinforce the importance of perceptual rendering while exposing content-specific factors. For textured meshes, *Textured Mesh Quality Assessment* [139] introduced a large-scale subjective dataset together with a deep learning-based metric, highlighting the interaction between geometry simplification, texture degradation, and semantic content in perceived quality. For dynamic digital humans, DDH-QA [140] established a dedicated quality assessment database covering both model-based and motion-based distortions, emphasizing that motion naturalness and animation artifacts are central to quality perception in human-centric 3D content.

In summary, projection-based methods better align objective modeling with the rendered perceptual interface actually seen by observers, but they are inherently shaped by viewpoint sampling and rendering design. Hybrid methods partially alleviate this limitation by reintroducing intrinsic geometric cues and therefore form an important bridge between pure rendering-based assessment and fully native 3D quality modeling.

### 4.4. Large Multimodal Model Approaches for 3DQA

LMM-based 3DQA explores how VLMs and MLLMs can introduce semantic grounding, language-conditioned reasoning, and explanation capability into 3DQA. Rather than forming two strictly separated categories, existing methods are better viewed as lying on a continuum: early approaches remain projection-primary and use rendered views as the main perceptual interface, while more recent models increasingly elevate native 3D representations to first-class inputs in multimodal reasoning.

At the projection-primary end, SJTU-H3D [48] introduced a subjective quality assessment benchmark for textured mesh digital humans together with a zero-shot NR evaluator. Its metric combines CLIP-based semantic affinity, low-level distortion cues from rendered projections, and lightweight mesh-geometry descriptors, showing that pretrained vision–language priors can improve semantic plausibility assessment beyond handcrafted distortion statistics. This line of work established an important precursor for language-aware 3D quality analysis, although the core perceptual evidence still comes from 2D renderings. LMM-PCQA [59] represents an early attempt to more explicitly integrate MLLMs into point-cloud quality assessment. It reformulates NR PCQA through text supervision by converting quality labels into natural-language descriptions, allowing the model to derive quality-related logits from rendered 2D projections. To alleviate the geometry loss introduced by rendering, it further incorporates multi-scale structural features extracted from the point cloud, yielding a hybrid projection-dominant yet structure-aware framework. BMPCQA [151] can be regarded as an LMM-based multimodal PCQA framework rather than a purely conventional deep model. It integrates rendering-projection, normal-image, and point-cloud patch features, and feeds them into a LMM for joint feature fusion and quality prediction. Compared with earlier projection-based quality regressors, this line of work highlights how multimodal language supervision can turn 3D quality evaluation into a richer perceptual reasoning problem with multi-task outputs.

At the native 3D-inclusive end, PIT-QMM [60] adopts an end-to-end point–image–text multimodal architecture for NR point-cloud quality assessment. By jointly consuming textual prompts, rendered image projections, and point-cloud inputs, it treats intrinsic 3D structure as a first-class modality rather than a lightweight auxiliary cue. This design enables the model to combine semantic context, projection-level appearance evidence, and native geometric structure within a unified multimodal pipeline, and also supports distortion localization and identification in addition to score prediction. More generally, large point-cloud language models such as PointLLM [152] suggest a broader foundation for future 3DQA. PointLLM [152] aligns point-cloud representations with LLM token spaces through large-scale pretraining and instruction tuning on point–text pairs. Although it was not originally designed for perceptual quality prediction, it provides a reusable 3D-language backbone that can potentially be adapted to quality labels, preference learning, or explanation-oriented supervision. From this perspective, PointLLM [152] is not a direct 3DQA solution, but it offers an important architectural prior for future explanation-capable quality assessment systems.

Overall, current LMM-based 3DQA methods evolve from projection-primary perceptual scoring toward more unified multimodal reasoning over rendered appearance, intrinsic geometry, and language. This trend suggests that future 3DQA systems may move beyond score regression alone and increasingly support explanation, distortion diagnosis, and interactive quality analysis.

To facilitate a concise comparison between representative conventional and LMM-based 3DQA methods, Table 9 summarizes their performance on three common NR point-cloud benchmarks under aligned within-dataset evaluation. Compared with the benchmark evidence in IQA and VQA, the current empirical basis in 3DQA is noticeably narrower, as directly comparable results are still concentrated on static point-cloud NR-PCQA rather than evenly covering meshes, digital humans, or dynamic 3D content. Within this more constrained setting, however, recent LMM-based methods already show clear competitiveness. In particular, PIT-QMM [60] achieves the best performance on all three benchmarks, while LMM-PCQA [59] also demonstrates strong results on LS-PCQA and WPC.

At the same time, the table suggests that the benefit of LMMs in 3DQA is not merely inherited from 2D quality scoring. Strong conventional baselines such as MM-PCQA [27] and CoPA+FT [149] remain highly competitive, indicating that point-cloud-specific representation design, multi-view rendering, and geometric sensitivity are still central to 3D quality prediction. Therefore, the current evidence supports a cautious but meaningful conclusion: LMM-based approaches have become a promising direction for NR point-cloud quality assessment, yet it remains premature to generalize this advantage to the broader 3DQA landscape without more comparable results on textured meshes, human-centric assets, and dynamic 4D content.

### 4.5. Summary of 3DQA Progression

Overall, the development of 3DQA reflects a transition from geometry-oriented comparison and projection-based quality estimation to increasingly hybrid and multimodal perceptual modeling. Early research mainly focused on FR geometric or projection-based metrics, while later NR methods introduced sparse 3D representations, multi-view rendering, and cross-modal fusion to better capture perceptual quality in point clouds and related 3D content.

As shown by the benchmark comparison above, recent LMM-based methods already demonstrate clear competitiveness on common NR point-cloud quality assessment benchmarks. In particular, MLLM-based approaches begin to outperform strong conventional baselines on LS-PCQA, SJTU-PCQA, and WPC, suggesting that multimodal reasoning and richer cross-view integration are becoming increasingly relevant to 3D perceptual assessment. At the same time, current evidence remains concentrated on static point-cloud settings, and comparable benchmark results for textured meshes, digital humans, and dynamic 3D content are still limited.

Therefore, the progression reviewed in this section is best understood as an expansion from geometry- and rendering-centric regression toward multimodal and potentially explanation-capable 3D quality modeling. The strongest empirical support at present lies in NR point-cloud quality assessment, while the broader applicability of LMMs across the full 3DQA spectrum remains an open and important direction for future study.

Before discussing cross-modal trends, we briefly summarize commonly used evaluation metrics and benchmarking protocols shared across these quality assessment tasks.

### 4.6. Evaluation Metrics and Benchmarking Protocols

In addition to benchmark datasets, evaluation metrics and protocols play a critical role in assessing the performance of visual quality assessment methods across image, video, and 3D modalities. Most existing approaches are evaluated according to their consistency with human subjective judgments, which are typically represented by MOS or differential MOS (DMOS). Commonly used metrics quantify different aspects of prediction performance, including rank consistency, linear correlation, and absolute error.

The Spearman rank-order correlation coefficient (SRCC) measures the monotonic relationship between predicted scores and subjective ratings:

(6) $$\mathrm{SRCC}=1-\frac{6\sum_{i=1}^{N}d_{i}^{2}}{N(N^{2}-1)},$$

where $d_{i}$ denotes the rank difference between the predicted score and the corresponding ground-truth score for the *i*th sample, and *N* is the total number of samples.

The Pearson linear correlation coefficient (PLCC) measures the linear correlation between predicted and subjective scores:

(7) $$\mathrm{PLCC}=\frac{\sum_{i=1}^{N}\left(x_{i}-\bar{x}\right)\left(y_{i}-\bar{y}\right)}{\sqrt{\sum_{i=1}^{N}{\left(x_{i}-\bar{x}\right)}^{2}}\sqrt{\sum_{i=1}^{N}{\left(y_{i}-\bar{y}\right)}^{2}}},$$

where $x_{i}$ and $y_{i}$ denote the predicted and ground-truth scores, respectively, and $\bar{x}$ and $\bar{y}$ are their sample means.

The Kendall rank correlation coefficient (KRCC) evaluates ranking consistency based on concordant and discordant pairs:

(8) $$\mathrm{KRCC}=\frac{N_{c}-N_{d}}{\frac{1}{2}N\left(N-1\right)},$$

where $N_{c}$ and $N_{d}$ are the numbers of concordant and discordant pairs, respectively.

The root mean square error (RMSE) reflects the average magnitude of prediction error:

(9) $$\mathrm{RMSE}=\sqrt{\frac{1}{N}\sum_{i=1}^{N}{\left(x_{i}-y_{i}\right)}^{2}}.$$

Among these metrics, the SRCC and KRCC mainly reflect ranking consistency, while the PLCC and RMSE emphasize score fidelity after regression or score alignment. In practice, the SRCC and PLCC are the most commonly reported criteria in IQA, VQA, and 3DQA benchmarks, whereas the KRCC and RMSE are often provided as complementary indicators.

Beyond metric selection, benchmarking protocols also substantially affect performance comparison. Common evaluation settings include random train–test splits within a dataset, cross-dataset testing, and leave-one-dataset-out validation for assessing generalization ability. For VQA, the temporal sampling strategy, clip duration, and sequence-level pooling can influence the final results. For 3DQA, viewpoint sampling, rendering settings, and multi-view aggregation protocols may also lead to different performance estimates.

The emergence of LMM-based methods further complicates evaluation. Recent approaches may generate textual explanations, pairwise preferences, or multi-dimensional attribute predictions instead of directly outputting scalar scores. As a result, future benchmarking should go beyond conventional correlation-based metrics and also consider explanation faithfulness, prediction consistency, robustness across prompts or datasets, and generalization across modalities.

## 5. Cross-Modal Trends and Unified Perspectives

Across image, video, and 3D modalities, visual quality assessment is undergoing a structural transformation that extends beyond increasing data dimensionality. More importantly, recent studies suggest that this transformation is not merely architectural but also conceptual: quality assessment is progressively shifting from distortion-centric regression toward semantically grounded, explainable, and context-conditioned evaluation.

Importantly, the motivation for adapting LMMs should not be reduced to using larger models for marginal benchmark gains alone. As shown by Table 3, Table 6, and Table 9, conventional and deep quality predictors remain highly competitive, especially on mature in-domain benchmarks. Nevertheless, many emerging scenarios—including AI-generated content, instruction-conditioned evaluation, comparative judgment, and explainable assessment—require capabilities that are difficult to express within a single scalar-regression formulation. In this sense, VLMs and MLLMs are better interpreted as extending the scope of quality assessment rather than uniformly replacing established predictors.

**Figure 2:**
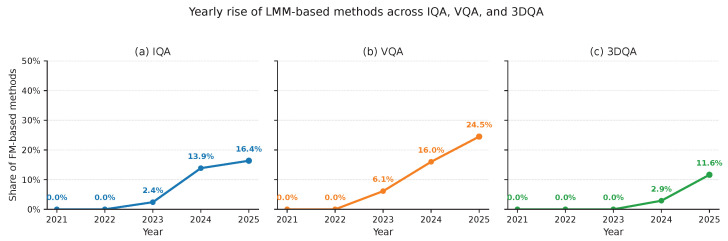
Yearly rise of LMM-based methods across IQA, VQA, and 3DQA. The statistics are derived from a keyword-based bibliographic survey of up to 1000 papers per domain, followed by cleaning and method-type categorization.

This broader shift is also reflected in the recent publication landscape. As shown in Figure 2, the share of large-/foundation-model-based methods rises steadily across all three domains but at clearly different rates. VQA exhibits the fastest growth, increasing from 0% in 2021–2022 to 6.1% in 2023, 16.0% in 2024, and 24.5% in 2025. IQA follows a more moderate trajectory, reaching 2.4%, 13.9%, and 16.4% over 2023–2025, while 3DQA starts later and remains smaller in scale, rising from 0% in 2021–2023 to 2.9% in 2024 and 11.6% in 2025. This asymmetry is broadly consistent with the benchmark evidence in this survey: in IQA, LMM-based methods mainly broaden the methodological space without yet showing uniformly dominant in-domain gains; in VQA, native LMM-based methods already exhibit clearer competitiveness on common UGC benchmarks; and in 3DQA, recent results are promising but still concentrated on a narrower NR point-cloud setting. The publication trend therefore suggests that the adoption of LMMs is task-driven rather than uniform, and appears strongest where long-context modeling, semantic reasoning, or multimodal fusion are most central to perceptual judgment.

First, the three modalities differ in how perceptual evidence is observed and integrated. IQA is based on fixed spatial observation, where judgments are formed from a single image. VQA introduces temporal accumulation, requiring models to account for motion consistency, temporal masking, and perceptual memory. 3DQA further depends on viewpoint-conditioned and rendering-mediated observation, where visibility, occlusion, shading, and projection strategy all affect perceived quality. These differences indicate that perceptual evidence becomes progressively more structured and condition-dependent across modalities.

Second, despite these modality-specific differences, a shared methodological progression can be observed. Conventional approaches in IQA, VQA, and 3DQA mostly formulate quality prediction as supervised regression toward MOS/DMOS, emphasizing distortion characterization and statistical correlation with human ratings. Recent LMM-based methods, however, increasingly move beyond direct score prediction. In IQA, methods such as DepictQA [52], DeQA-Score [91], and Grounding-IQA [54] demonstrate that quality evaluation can be coupled with descriptive reasoning, score distribution modeling, and region-aware grounding. In the video domain, CP-LLM [58], ${\mathrm{VQA}}^{2}$ [123], and VQAThinker [122] further extend quality assessment toward description generation, question answering, and reasoning-aided interpretation. For 3D content, LMM-PCQA [59] and PIT-QMM [60] show that projection-based evidence and intrinsic 3D structure can be jointly incorporated into multimodal quality reasoning. These studies collectively suggest that quality assessment is evolving from black-box regression into a more explainable perceptual inference process.

Third, perceptual quality itself is increasingly defined by semantic plausibility and structural coherence rather than only low-level artifact visibility. This trend is especially evident in AI-generated and enhancement-oriented scenarios. Datasets such as AIGIQA-20K [29], PKU-AIGIQA-4K [74], and HVEval [35] reveal that human judgments often depend on realism, alignment, and consistency beyond conventional distortions. In human-centered settings, AGHI-QA [76] and FVQ [124] further highlight identity preservation, motion plausibility, and structural consistency as essential perceptual factors. These developments indicate that future quality assessment systems must integrate distortion sensitivity with semantic understanding and high-level structural reasoning.

Fourth, recent work also points to a gradual transition from modality-specific predictors toward unified multimodal quality assessment models. For instance, Q-Align [129] demonstrates that a single LMM can be trained to handle IQA, image aesthetic assessment (IAA), and VQA within a unified framework by leveraging text-defined rating levels. Q-Align also shows that jointly learning across IQA and VQA datasets can improve both accuracy and generalization, especially under mixed-data scenarios. Together with the publication-trend analysis in Figure 2, these findings suggest that quality assessment is increasingly moving toward a unified multimodal reasoning paradigm, although the pace of this transition still differs across image, video, and 3D domains.

## 6. Conclusions and Future Outlook

Visual quality assessment is undergoing a fundamental transformation driven by both the evolution of visual sensing modalities and the emergence of LMMs. From spatial observation in images to temporal integration in videos and viewpoint-conditioned perception in 3D content, each modality introduces new challenges in modeling perceptual evidence. Meanwhile, the field is shifting from distortion-centric regression toward semantically grounded and reasoning-capable evaluation paradigms.

Taken together, the benchmark comparisons and publication-trend analysis in this survey suggest that the rationale for adapting LMMs is not uniform across modalities. In IQA, where standard in-domain benchmarks are already highly mature, the present value of LMM-based methods lies more in promptable, explainable, and semantically grounded assessment than in uniformly superior correlation scores. In VQA, the joint modeling of spatial distortion, motion dynamics, and temporal coherence makes LMM-based approaches more directly competitive, which is also reflected in their recent faster growth. In 3DQA, early evidence on the NR point-cloud quality assessment is promising, but the empirical basis remains narrower than in image and video settings. Therefore, the adoption of LMMs should be understood as a task-dependent methodological transition rather than a universal replacement of conventional quality predictors.

This survey presents a unified perspective on quality assessment across image, video, and 3D modalities. By reviewing datasets, conventional approaches, and recent LMM-based methods, we show that quality assessment is evolving from a static signal-to-score mapping to a multimodal perceptual reasoning process, where predictions can be conditioned on textual criteria and supported by explanatory evidence.

Building upon these observations, we outline several key research directions:

(1) Unified cross-modal perceptual representation. Learning representations that generalize across image, video, and 3D modalities remains a fundamental challenge. Future work should jointly model spatial structure, temporal dynamics, and viewpoint-dependent perception within a unified framework.
(2) Reliable and interpretable score calibration and benchmarking. LMM-based methods often produce textual, comparative, or multi-attribute outputs instead of scalar scores. Establishing consistent and interpretable mappings to quantitative quality measures, while also designing fair benchmarking protocols across prompts, datasets, and modalities, is essential for rigorous evaluation and practical deployment.
(3) Long-context modeling for VQA. Video quality depends on long temporal dependencies involving motion consistency and temporal evolution. Although recent methods improve contextual reasoning, efficient and scalable long-context modeling remains largely underexplored.
(4) Explainable quality assessment. LMM-based approaches enable textual explanations alongside quality predictions. However, ensuring the faithfulness and reliability of such explanations remains challenging, especially in aligning them with perceptual evidence.
(5) Grounded quality assessment with spatial and structural alignment. Grounding quality judgments in spatial regions, temporal segments, or geometric structures is still underexplored. Improving localization accuracy and cross-modal consistency is essential for trustworthy assessment.

Overall, visual quality assessment is converging toward a unified multimodal paradigm that integrates distortion sensitivity, semantic understanding, temporal reasoning, and viewpoint-aware perception. Advancing this paradigm requires not only larger models but also principled evaluation frameworks and deeper integration of perceptual theory.

In this sense, future visual quality assessment systems may evolve from isolated evaluators into general multimodal perceptual intelligence systems. 

## Figures and Tables

**Table 1:** Representative quality assessment datasets for images.

Dataset	Year	Scale	Models	Description
Natural IQA Datasets				
LIVE [61]	2004	779	–	Classical IQA benchmark with compression/blur/noise.
TID2008 [62]	2008	1700	–	FR-IQA; 17 distortion types with reference images.
TID2013 [63]	2013	3000	–	FR-IQA; 24 distortion types with references.
MDID2016 [64]	2016	1600	–	Random distortion types and severity levels.
DIBR [65]	2011	84	–	DIBR-rendered views with synthesis artifacts.
SIQAD [66]	2014	980	–	Screen-content images; text and graphics distortions.
SCIQ [67]	2017	1800	–	Screen-content IQA; 9 distortion types.
KADID-10K [68]	2019	10,125	–	Synthetic distortions; 24 common types.
KonIQ-10k [2]	2018	10,073	–	In-the-wild images (YFCC100m) with authentic artifacts.
UHD-IQA [69]	2024	6073	–	UHD/4K photos.
AI-generated IQA Datasets				
DiffusionDB [70]	2022	14M	1	Large-scale text-to-image generations; no quality labels.
HPS [71]	2023	98.8K	1	Human preference supervision.
ImageReward [72]	2023	136.9K	3	Preference-based reward modeling.
Pick-A-Pic [73]	2023	500K	6	Pairwise human preference dataset.
AGIQA-3K [28]	2023	2982	6	Perceptual quality + alignment.
AIGIQA-20K [29]	2024	20K	15	Large-scale perceptual quality + alignment.
PKU-AIGIQA-4K [74]	2025	4K	3	Text-to-image + image-to-image perceptual quality.
AIGI-VC [75]	2025	2500	5	Visual communication quality.
AGHI-QA [76]	2025	4000	10	AI-generated human images quality.

**Table 2:** Comparison of representative IQA approaches.

Method	Year	Output	Paradigm	Key Characteristics
Conventional IQA Methods				
SSIM [6]	2004	Score	Handcrafted	Structural similarity; interpretable signal-fidelity modeling.
BRISQUE [11]	2012	Score	NSS-based	Natural scene statistics features for blind distortion-sensitive prediction.
CNNIQA [13]	2014	Score	CNN	Patch-based end-to-end quality regression.
NIMA [14]	2017	Distribution	CNN	Predicts rating distributions to model subjective variability.
DBCNN [15]	2019	Score	CNN	Bilinear fusion of content-aware and distortion-aware features.
HyperIQA [16]	2020	Score	CNN	Hyper-network generates image-adaptive regression parameters.
UNIQUE [17]	2021	Score	Unsupervised deep	Uncertainty-aware quality estimation with unsupervised feature learning.
StairIQA [18]	2023	Score	Deep mixed-training	Iterative mixed-dataset training for stronger cross-database generalization.
PromptIQA [49]	2024	Score	Prompt-conditioned	Requirement-aware quality scoring from image-score demonstrations.
ELIQ [30]	2026	Score	Label-free generative	AI-IQA via distributional and evolutionary consistency.
LMM-based IQA Methods				
CLIP-IQA [42]	2022	Score	VLM	Zero-shot prompt-based image–text alignment mapped to scalar quality.
QA-CLIP [43]	2023	Score	VLM-tuned	Learnable prompt tuning for finer quality-level discrimination.
QualiCLIP [89]	2024	Score	VLM self-supervised	Monotonic degradation ranking for label-efficient quality-aware alignment.
LIQE [44]	2023	Score	VLM multi-task	Joint modeling of quality, scene, and distortion attributes.
ZEN-IQA [45]	2024	Score + Text	Zero-shot VLM	Explainable NR-IQA via quality prompts without IQA training.
2AFC [50]	2024	Score	MLLM reasoning	Pairwise preference reasoning aggregated into global quality scores.
Compare2Score [51]	2024	Score	MLLM reasoning	Structured relative judgments converted into scalar quality scores.
DepictQA [52]	2024	Score + Text	MLLM explainable	Structured critique, comparison, and diagnostic feedback.
Q-Instruct [53]	2024	Score	Instruction-tuned MLLM	Low-level visual instruction tuning for stronger distortion sensitivity.
Grounding-IQA [54]	2024	Score + Text	Grounded MLLM	Region-aware quality reasoning with localized explanatory evidence.
iDETEX [90]	2025	Score + Text	Grounded MLLM	Joint scoring, defect localization, and detailed critique generation.
DeQA-Score [91]	2025	Distribution	MLLM explainable	Distribution-based quality regression in the DepictQA framework.
DepictQA-Wild [92]	2025	Score + Text	MLLM explainable	In-the-wild descriptive IQA with assessment and comparison.

**Table 3:** Performance comparison of representative conventional and LMM-based IQA methods on common benchmarks. Results are reported as SRCC/PLCC, and the best results in each column are shown in bold.

Method	Year	Category	Synthetic Distortion			Authentic Distortion		
			LIVE	CSIQ	KADID-10K	BID	CLIVE	KonIQ-10k
HyperIQA	2020	CNN	0.950/0.952	0.938/0.951	0.849/0.851	0.866/0.880	0.851/0.872	0.911/0.922
MANIQA	2022	Transformer	0.956/0.960	0.937/0.948	0.921/0.919	0.901/0.910	0.892/0.913	0.929/**0.945**
MoNet	2023	CNN/Transformer hybrid	0.956/0.960	**0.955**/**0.961**	0.921/0.923	0.901/0.915	0.900/0.917	0.928/**0.945**
StairIQA	2023	Mixed-training NR-IQA	0.937/0.934	0.768/0.843	0.785/0.805	0.774/0.788	0.780/0.855	0.865/0.896
PromptIQA	2024	Prompt-based NR-IQA	0.936/0.934	0.926/0.939	0.928/0.931	0.915/0.934	0.913/**0.928**	0.929/0.943
LIQE	2023	VLM	0.970/0.951	0.936/0.939	0.930/0.931	0.875/0.900	0.904/0.910	0.919/0.908
Q-Align	2024	MLLM	0.913/0.919	0.915/0.936	0.869/0.927	0.904/0.920	**0.931**/0.921	**0.935**/0.934
Compare2Score	2024	MLLM	**0.972**/**0.969**	0.950/0.943	**0.952**/**0.939**	**0.919**/**0.939**	0.914/**0.928**	0.931/0.939

**Table 4:** Representative quality assessment datasets for videos.

Dataset	Year	Scale	Models	Description
Natural VQA Datasets				
LIVE-VQA [95]	2008	160	–	Natural videos; compression and packet-loss distortions.
CVD2014 [96]	2014	234	–	Consumer videos from diverse cameras and pipelines.
LIVE-Qualcomm [97]	2016	208	–	Mobile artifacts: focus, exposure, motion, and low-light.
KoNViD-1k [98]	2017	1200	–	In-the-wild UGC (YFCC100m); authentic distortions.
LIVE-VQC [99]	2018	585	–	User-captured videos under diverse conditions.
YouTube-UGC [100]	2019	1380	–	Varied content with realistic artifacts.
LSVQ [101]	2021	39,075	–	Large-scale dataset with real-world distortions.
LIVE-NFLX-II [102]	2018	588	–	Streaming videos; wide bitrate and rebuffering scenarios.
SQoE-IV [103]	2019	1450	–	Streaming quality of experience evaluation.
FineVD [104]	2025	6104	–	Fine-grained UGC VQA; multi-attribute annotations with rationales.
CG-VQD [105]	2025	75	–	Rendered CG videos; aliasing and denoiser flicker artifacts.
SCD [106]	2025	1600	–	Screen-content VQA; UI/text and compression artifacts.
MVQA-68K [107]	2025	68,000	–	Multi-dimensional and interpretable VQA.
AI-generated VQA Datasets				
FETV [32]	2023	2476	4	Fine-grained fidelity + temporal QA.
VBench [33]	2023	6984	4	Multi-aspect video benchmark.
MQT [108]	2023	1005	4	Semantic + visual fidelity QA.
EvalCrafter [34]	2024	5600	8	Holistic video generation evaluation.
TC-Bench [109]	2024	150	3	Temporal compositionality benchmark.
DEVIL [110]	2025	800	3	Dynamicity-focused evaluation.
T2V-CompBench [111]	2025	700	3	Compositional video benchmark.
HVEval [35]	2025	20,000	24	Human-centric AIGV.

**Table 5:** Comparison of representative VQA approaches.

Method	Year	Output	Paradigm	Key Characteristics
***Conventional VQA Methods***				
MT-STQA [25]	2009	Score	FR-HVS	Motion-aware spatio-temporal human-visual-system modeling.
STRRED [113]	2013	Score	RR-NSS	Reduced-reference entropic difference with compact statistics.
Video BLIINDS [19]	2014	Score	NR-NSS	Spatio-temporal NSS with motion coherence for blind VQA.
VSFA [21]	2019	Score	CNN+RNN	CNN features with GRU-based temporal memory modeling.
FAST-VQA [26]	2022	Score	Transformer	Fragment sampling with attention aggregation for efficient VQA.
DisCoVQA [117]	2023	Score	Transformer	Distortion–content interaction modeling via temporal coupling.
STI-VQA [118]	2023	Score	Transformer	Spatial-motion interaction using tokenized distortion and motion cues.
DOVER [120]	2023	Score	Dual-branch Transformer	Disentangles technical quality perception from aesthetic preference.
ModularBVQA [121]	2024	Score	Deep modular	Resolution- and frame-rate-aware rectifiers for format variability.
FineVQ [104]	2025	Score	Deep supervised	Fine-grained attribute ratings with degradation-type annotations.
***LMM-based VQA Methods***				
BUONAVISTA [46]	2023	Score	VLM	Opinion-unaware prompt alignment fused with technical quality cues.
BVQI [47]	2023	Score	VLM	Localized prompt alignment for semantically aware failure analysis.
LMM-VQA [57]	2025	Score + Class	MLLM	Instruction-conditioned QA-style MOS prediction from spatio-temporal tokens.
CP-LLM [58]	2025	Score + Text	MLLM	Dual-encoder reasoning for scoring, description, and comparison.
VQAThinker [122]	2025	Score + Text	MLLM (RL)	RL-based interpretable distortion analysis and robust score prediction.
${\mathrm{VQA}}^{2}$ [123]	2025	Score + QA	MLLM	Interleaved visual–motion tokens for scoring and quality-related QA.
FVQ [124]	2025	Score	MLLM	Face-centric quality modeling with identity, detail, and temporal fidelity.

**Table 6:** Performance comparison of representative conventional and LMM-based VQA methods on five common UGC benchmarks under as closely aligned an in-domain setting as possible. Results are reported as SRCC/PLCC, the best results in each column are shown in bold, and “–” indicates that a directly comparable result was not available from the corresponding paper under the aligned protocol.

Method	Year	Category	LSVQ-Test	LSVQ-1080p	KoNViD-1k	LIVE-VQC	YouTube-UGC
NIQE [12]	2013	NSS-based	0.442/0.332	0.489/0.459	0.541/0.553	0.596/0.628	0.278/0.290
VIIDEO [114]	2015	Unsupervised	0.080/0.080	0.009/0.019	0.299/0.300	0.033/0.215	0.058/0.154
STEM [115]	2022	Classical NR-VQA	0.206/0.243	0.434/0.381	0.619/0.627	0.594/0.629	0.284/0.318
FAST-VQA [26]	2022	Transformer	0.880/0.880	0.781/0.813	0.859/0.854	0.826/0.845	0.730/0.747
DOVER [120]	2023	Dual-branch Transformer	0.878/0.866	0.782/0.813	0.874/0.869	0.817/0.840	0.771/0.781
SAMA [119]	2024	Attention-based deep model	0.883/0.884	0.782/0.822	0.880/0.877	0.834/0.849	–
Memory-VQA [128]	2025	Memory-inspired deep model	0.897/0.895	0.807/0.841	0.921/0.923	0.866/0.887	0.891/0.885
Q-Align [129]	2024	MLLM	0.886/0.884	0.761/0.822	0.876/0.878	0.783/0.819	0.834/0.846
LMM-VQA [57]	2025	MLLM	**0.913/0.914**	**0.879/0.898**	**0.929/0.930**	**0.891/0.903**	**0.901/0.897**
VQA2 (UGC-Scorer) [123]	2025	MLLM	0.878/0.872	0.794/0.821	0.881/0.880	0.785/0.830	0.811/0.823
Q-Insight [93]	2025	Reasoning-based MLLM	0.644/0.639	0.601/0.648	0.751/0.753	0.624/0.708	0.560/0.591
VisualQuality-R1 [94]	2025	RL-based MLLM	0.795/0.796	0.716/0.744	0.784/0.792	0.732/0.781	0.717/0.730
VQAThinker [122]	2025	MLLM	0.883/0.880	0.798/0.834	0.881/0.884	0.808/0.847	0.860/0.863
VQ-Insight [127]	2026	Reasoning-based MLLM	0.875/0.876	0.786/0.823	0.875/0.884	0.790/0.835	–

**Table 7:** Representative quality assessment datasets for 3D content.

Dataset	Year	Scale	Description
G-PCD [130,131]	2017	40	Geometry-only point clouds; geometric distortions.
RG-PCD [132]	2018	24	Geometry-only point clouds with diverse contents.
VsenseVVDB [133]	2019	32	Volumetric videos; compression artifacts.
IRPC [134]	2020	108	Point clouds; rendering and coding pipeline variations.
WPC [135]	2021	740	Point clouds; compression, downsampling, and Gaussian noise.
WPC2.0 [136]	2021	400	Compressed point-cloud sequences; temporal artifacts.
SJTU-PCQA [23]	2020	420	Point clouds; common distortions for benchmarking.
LS-PCQA [3]	2023	1080	NR-PCQA; realistic distortions without references.
BASICS [137]	2023	1494	Static point clouds; broad content coverage.
CMDM [138]	2020	80	Colored meshes; geometry and texture degradations.
TMQA [139]	2023	3000	Textured meshes; controlled distortions.
DDHQA [140]	2023	800	Dynamic digital humans; temporal mesh and texture artifacts.
SJTU-H3D [48]	2023	1120	Static digital humans; geometry, texture, and rendering artifacts.
DPCD [141]	2025	525	Dynamic point clouds; compression and temporal noise.
DHQA-4D [142]	2025	1920	Dynamic 4D humans; textured temporal artifacts.

**Table 8:** Comparison of representative 3DQA approaches. The terms 2D, 3D, and text describe the primary input modalities: **2D** refers to rendered projections that approximate human-visual perception, **3D** refers to native geometric and attribute representations (e.g., point clouds or meshes), and **text** denotes language-based inputs such as prompts or instructions used in multimodal reasoning.

Method	Year	Output	Paradigm	Key Characteristics
***Conventional 3DQA Methods***				
PCQM [22]	2020	Score	FR (3D)	Point-to-surface + quadric fitting; geometry–color joint metric.
Point2Dist [144]	2021	Score	FR (3D)	Local geometry/color distributions with statistical distance.
MS-GraphSIM [143]	2021	Score	FR (3D)	Multi-scale graph similarity on structure and color gradients.
VisionTasks-PCQA [145]	2020	Score	Multi-task (3D)	Auxiliary vision-task degradation as perceptual proxy.
ResSCNN [3]	2023	Score	NR (3D)	Sparse CNN learns hierarchical quality-aware point features.
SJTU-PCQA [23]	2020	Score	FR (2D)	Multi-view rendering with cube-face aggregation.
PQA-Net [24]	2021	Score	NR (2D)	View-wise CNN encoding with learned aggregation.
IT-PCQA [146]	2022	Score	Transfer (2D)	Domain adaptation from 2D IQA via multi-view images.
GMS-3DQA [147]	2023	Score	Efficient (2D)	Gradient-based mini-patch sampling for redundancy reduction.
MovingCam-PCQA [148]	2023	Score	Sequential (2D)	Moving-camera rendering with temporal feature fusion.
MM-PCQA [27]	2023	Score	Hybrid (2D+3D)	Cross-modal attention over 3D geometry and projections.
CoPA [149]	2024	Score	Hybrid (2D+3D)	Contrastive pretraining with semantic-guided multi-view fusion.
MPV-PCQA [150]	2025	Score	Hybrid (2D+3D)	Joint modeling of point clouds and dynamic video streams.
***LMM-Based 3DQA Methods***				
SJTU-H3D [48]	2023	Score	VLM (2D)	CLIP-based semantic affinity on rendered views.
LMM-PCQA [59]	2024	Score+Text	MLLM (2D+3D+Text)	Instruction-tuned reasoning with projection + structure cues.
BMPCQA [151]	2025	Score	MLLM (2D+3D+Text)	Large multimodal fusion of projections, normals, and point patches.
PointLLM [152]	2024	Text	3D-LLM (3D+Text)	Point-cloud–language alignment via instruction tuning.
PIT-QMM [60]	2025	Score+Text	MLLM (2D+3D+Text)	Unified point–image–text fusion with reasoning capability.

**Table 9:** Performance comparison of representative conventional and LMM-based NR-PCQA methods on three common point-cloud benchmarks under aligned within-dataset evaluation. Results are reported as SRCC/PLCC, and the best results in each column are shown in bold. To avoid conflating fundamentally different protocols, this table focuses on NR point-cloud quality assessment rather than mixing FR metrics or heterogeneous 3D content types.

Method	Year	Category	LS-PCQA	SJTU-PCQA	WPC
IT-PCQA [146]	2022	Domain adaptation	0.326/0.347	0.539/0.629	0.422/0.468
ResSCNN [3]	2023	Sparse CNN	0.594/0.624	0.834/0.863	0.735/0.752
MM-PCQA [27]	2023	Hybrid deep model	0.581/0.597	0.876/0.898	0.761/0.774
CoPA+FT [149]	2024	Contrastive pretraining	0.613/0.636	0.897/0.913	0.779/0.785
LMM-PCQA [59]	2024	MLLM-assisted	0.684/0.691	0.730/0.724	0.854/0.825
PIT-QMM [60]	2025	MLLM	**0.751/0.766**	**0.906/0.916**	**0.872/0.844**

## Data Availability

No new data were created or analyzed in this study. Data sharing is not applicable to this article.

## References

[1] Fiedler M., Hossfeld T., Tran-Gia P. (2010). A generic quantitative relationship between quality of experience and quality of service. IEEE Netw..

[2] Hosu V., Lin H., Sziranyi T., Saupe D. (2020). KonIQ-10k: An ecologically valid database for deep learning of blind image quality assessment. IEEE Trans. Image Process..

[3] Liu Y., Yang Q., Xu Y., Yang L. (2023). Point cloud quality assessment: Dataset construction and learning-based no-reference metric. ACM Trans. Multimed. Comput. Commun. Appl..

[4] Series B. (2012). Methodology for the subjective assessment of the quality of television pictures. Recommendation ITU-R BT.

[5] Installations T., Line L. (1999). Subjective video quality assessment methods for multimedia applications. Networks.

[6] Wang Z., Bovik A.C., Sheikh H.R., Simoncelli E.P. (2004). Image quality assessment: From error visibility to structural similarity. IEEE Trans. Image Process..

[7] Wang Z., Simoncelli E.P., Bovik A.C. (2003). Multiscale structural similarity for image quality assessment. Proceedings of the Thirty-Seventh Asilomar Conference on Signals, Systems & Computers, Pacific Grove, CA, USA, 9–12 November 2003.

[8] Sheikh H.R., Bovik A.C. (2006). Image information and visual quality. IEEE Trans. Image Process..

[9] Zhang L., Zhang L., Mou X., Zhang D. (2011). FSIM: A feature similarity index for image quality assessment. IEEE Trans. Image Process..

[10] Xue W., Zhang L., Mou X., Bovik A.C. (2013). Gradient magnitude similarity deviation: A highly efficient perceptual image quality index. IEEE Trans. Image Process..

[11] Mittal A., Moorthy A.K., Bovik A.C. (2012). No-reference image quality assessment in the spatial domain. IEEE Trans. Image Process..

[12] Mittal A., Soundararajan R., Bovik A.C. (2012). Making a “completely blind” image quality analyzer. IEEE Signal Process. Lett..

[13] Kang L., Ye P., Li Y., Doermann D. Convolutional neural networks for no-reference image quality assessment. Proceedings of the IEEE Conference on Computer Vision and Pattern Recognition.

[14] Talebi H., Milanfar P. (2018). NIMA: Neural image assessment. IEEE Trans. Image Process..

[15] Zhang W., Ma K., Yan J., Deng D., Wang Z. (2018). Blind image quality assessment using a deep bilinear convolutional neural network. IEEE Trans. Circuits Syst. Video Technol..

[16] Su S., Yan Q., Zhu Y., Zhang C., Ge X., Sun J., Zhang Y. Blindly assess image quality in the wild guided by a self-adaptive hyper network. Proceedings of the IEEE/CVF Conference on Computer Vision and Pattern Recognition.

[17] Zhang W., Ma K., Zhai G., Yang X. (2021). Uncertainty-aware blind image quality assessment in the laboratory and wild. IEEE Trans. Image Process..

[18] Sun W., Min X., Tu D., Ma S., Zhai G. (2023). Blind quality assessment for in-the-wild images via hierarchical feature fusion and iterative mixed database training. IEEE J. Sel. Top. Signal Process..

[19] Saad M.A., Bovik A.C., Charrier C. (2014). Blind Prediction of Natural Video Quality. IEEE Trans. Image Process..

[20] Korhonen J. (2019). Two-Level Approach for No-Reference Consumer Video Quality Assessment. IEEE Trans. Image Process..

[21] Li D., Jiang T., Jiang M. Quality assessment of in-the-wild videos. Proceedings of the 27th ACM International Conference on Multimedia.

[22] Nehme Y., Dupont F., Lavou’e G., Le Callet P. PCQM: A Full-Reference Quality Metric for Colored 3D Point Clouds. Proceedings of the 2020 Twelfth International Conference on Quality of Multimedia Experience (QoMEX).

[23] Yang Q., Chen H., Ma Z., Xu Y., Tang R., Sun J. (2020). Predicting the perceptual quality of point cloud: A 3d-to-2d projection-based exploration. IEEE Trans. Multimed..

[24] Liu Q., Yuan H., Su H., Liu H., Wang Y., Yang H., Hou J. (2021). PQA-Net: Deep No Reference Point Cloud Quality Assessment via Multi-view Projection. IEEE Trans. Circuits Syst. Video Technol..

[25] Seshadrinathan K., Bovik A.C. (2009). Motion tuned spatio-temporal quality assessment of natural videos. IEEE Trans. Image Process..

[26] Wu H., Chen C., Hou J., Liao L., Wang A., Sun W., Yan Q., Lin W. Fast-VQA: Efficient End-to-End Video Quality Assessment with Fragment Sampling. Proceedings of the European Conference on Computer Vision (ECCV).

[27] Zhang Z., Sun W., Min X., Zhou Q., He J., Wang Q., Zhai G. (2022). Mm-pcqa: Multi-modal learning for no-reference point cloud quality assessment. arXiv.

[28] Li C., Zhang Z., Wu H., Sun W., Min X., Liu X., Zhai G., Lin W. (2023). Agiqa-3k: An open database for ai-generated image quality assessment. IEEE Trans. Circuits Syst. Video Technol..

[29] Li C., Kou T., Gao Y., Cao Y., Sun W., Zhang Z., Zhou Y., Zhang Z., Zhang W., Wu H. Aigiqa-20k: A large database for ai-generated image quality. Proceedings of the IEEE/CVF Conference on Computer Vision and Pattern Recognition Workshops.

[30] Li X., Xu Z., Zhang Z., Cai Z., Wu S., Min X., Chen Y., Zhai G. (2026). ELIQ: A Label-Free Framework for Quality Assessment of Evolving AI-Generated Images. arXiv.

[31] Han S., Fan H., Kong F., Liao W., Guo C., Li C., Timofte R., Li L., Li T., Cui J. NTIRE 2025 challenge on text to image generation model quality assessment. Proceedings of the IEEE/CVF Conference on Computer Vision and Pattern Recognition (CVPR) Workshops.

[32] Liu Y., Li L., Ren S., Gao R., Li S., Chen S., Sun X., Hou L. (2024). Fetv: A benchmark for fine-grained evaluation of open-domain text-to-video generation. Adv. Neural Inf. Process. Syst..

[33] Huang Z., He Y., Yu J., Zhang F., Si C., Jiang Y., Zhang Y., Wu T., Jin Q., Chanpaisit N. VBench: Comprehensive Benchmark Suite for Video Generative Models. Proceedings of the IEEE/CVF Conference on Computer Vision and Pattern Recognition.

[34] Liu Y., Cun X., Liu X., Wang X., Zhang Y., Chen H., Liu Y., Zeng T., Chan R., Shan Y. Evalcrafter: Benchmarking and evaluating large video generation models. Proceedings of the IEEE/CVF Conference on Computer Vision and Pattern Recognition.

[35] Wu S., Li Y., Duan H., Jiang Y., Zhu Y., Zhai G. Hveval: Towards unified evaluation of human-centric video generation and understanding. Proceedings of the 33rd ACM International Conference on Multimedia.

[36] Radford A., Kim J.W., Hallacy C., Ramesh A., Goh G., Agarwal S., Sastry G., Askell A., Mishkin P., Clark J. Learning transferable visual models from natural language supervision. Proceedings of the International Conference on Machine Learning.

[37] Alayrac J.B., Recasens A., Schneider R., Arandjelovic R., Ramapuram J., De Fauw J., Smaira L., Dieleman S., Zisserman A. Flamingo: A visual language model for few-shot learning. Proceedings of the Advances in Neural Information Processing Systems (NeurIPS).

[38] Yang Z., Li L., Lin K., Wang J., Lin C.C., Liu Z., Wang L. (2023). The dawn of lmms: Preliminary explorations with gpt-4v (ision). arXiv.

[39] Liu H., Li C., Wu Q., Lee Y.J. (2023). Visual Instruction Tuning. arXiv.

[40] Wang P., Bai S., Tan S., Wang S., Fan Z., Bai J., Chen K., Liu X., Wang J., Ge W. (2024). Qwen2-vl: Enhancing vision-language model’s perception of the world at any resolution. arXiv.

[41] Li J., Li D., Savarese S., Hoi S. BLIP-2: Bootstrapping Language-Image Pre-training with Frozen Image Encoders and Large Language Models. Proceedings of the 40th International Conference on Machine Learning.

[42] Wang J., Chan K.C., Loy C.C. (2023). Exploring CLIP for Assessing the Look and Feel of Images. Proc. AAAI Conf. Artif. Intell..

[43] Pan W., Yang Z., Liu D., Fang C., Zhang Y., Dai P. (2023). Quality-Aware CLIP for Blind Image Quality Assessment. Proceedings of the Pattern Recognition and Computer Vision (PRCV 2023); Lecture Notes in Computer Science.

[44] Zhang W., Zhai G., Wei Y., Yang X., Ma K. Blind Image Quality Assessment via Vision-Language Correspondence: A Multitask Learning Perspective. Proceedings of the IEEE/CVF Conference on Computer Vision and Pattern Recognition (CVPR).

[45] Miyata T. (2024). ZEN-IQA: Zero-Shot Explainable and No-Reference Image Quality Assessment with Vision Language Model. IEEE Access.

[46] Wu H., Liao L., Hou J., Chen C., Zhang E., Wang A., Sun W., Yan Q., Lin W. (2023). Exploring Opinion-unaware Video Quality Assessment with Semantic Affinity Criterion. arXiv.

[47] Wu H., Liao L., Wang A., Chen C., Hou J., Sun W., Yan Q., Lin W. (2023). Towards Robust Text-Prompted Semantic Criterion for In-the-Wild Video Quality Assessment. arXiv.

[48] Zhang Z., Sun W., Zhou Y., Wu H., Li C., Min X., Liu X., Zhai G., Lin W. (2025). Advancing Zero-Shot Digital Human Quality Assessment Through Text-Prompted Evaluation. IEEE Trans. Image Process..

[49] Chen Z., Qin H., Wang J., Yuan C., Li B., Hu W., Wang L. PromptIQA: Boosting the Performance and Generalization for No-Reference Image Quality Assessment via Prompts. Proceedings of the European Conference on Computer Vision (ECCV).

[50] Zhu H., Sui X., Chen B., Liu X., Chen P., Fang Y., Wang S. (2024). 2AFC prompting of large multimodal models for image quality assessment. IEEE Trans. Circuits Syst. Video Technol..

[51] Zhu H., Wu H., Li Y., Zhang Z., Chen B., Zhu L., Fang Y., Zhai G., Lin W., Wang S. Adaptive Image Quality Assessment via Teaching Large Multimodal Model to Compare. Proceedings of the Advances in Neural Information Processing Systems.

[52] You Z., Li Z., Gu J., Yin Z., Xue T., Dong C. Depicting Beyond Scores: Advancing Image Quality Assessment through Multi-Modal Language Models. Proceedings of the European Conference on Computer Vision (ECCV).

[53] Wu H., Zhang Z., Zhang E., Chen C., Liao L., Wang A., Xu K., Li C., Hou J., Zhai G. Q-Instruct: Improving Low-level Visual Abilities for Multi-modality Foundation Models. Proceedings of the IEEE/CVF Conference on Computer Vision and Pattern Recognition (CVPR).

[54] Chen Z., Zhang X., Li W., Pei R., Song F., Min X., Liu X., Yuan X., Guo Y., Zhang Y. (2024). Grounding-iqa: Multimodal language grounding model for image quality assessment. arXiv.

[55] Wu H., Zhang Z., Zhang E., Chen C., Liao L., Wang A., Li C., Sun W., Yan Q., Zhai G. (2023). Q-bench: A benchmark for general-purpose foundation models on low-level vision. arXiv.

[56] Huang Z., Zhang Z., Lu Y., Zha Z.J., Chen Z., Guo B. (2024). Visualcritic: Making lmms perceive visual quality like humans. arXiv.

[57] Ge Q., Sun W., Zhang Y., Li Y., Ji Z., Sun F., Jui S., Min X., Zhai G. (2025). LMM-VQA: Advancing Video Quality Assessment with Large Multimodal Models. IEEE Trans. Circuits Syst. Video Technol..

[58] Wen W., Wu Y., Sheng Y., Birkbeck N., Adsumilli B., Wang Y. (2025). CP-LLM: Context and Pixel Aware Large Language Model for Video Quality Assessment. arXiv.

[59] Zhang Z., Wu H., Zhou Y., Li C., Sun W., Chen C., Min X., Liu X., Lin W., Zhai G. (2024). LMM-PCQA: Assisting Point Cloud Quality Assessment with LMM. Proceedings of the 32nd ACM International Conference on Multimedia, 2024.

[60] Gupta S., Phillips G., Bovik A.C. (2025). PIT-QMM: A Large Multimodal Model for No-Reference Point Cloud Quality Assessment. Proceedings of the 2025 IEEE International Conference on Image Processing (ICIP).

[61] Sheikh H. (2005). LIVE Image Quality Assessment Database Release 2. http://live.ece.utexas.edu/research/quality.

[62] Ponomarenko N., Lukin V., Zelensky A., Egiazarian K., Carli M., Battisti F. (2009). TID2008-a database for evaluation of full-reference visual quality assessment metrics. Adv. Mod. Radioelectron..

[63] Ponomarenko N., Jin L., Ieremeiev O., Lukin V., Egiazarian K., Astola J., Vozel B., Chehdi K., Carli M., Battisti F. (2015). Image database TID2013: Peculiarities, results and perspectives. Signal Process. Image Commun..

[64] Sun W., Zhou F., Liao Q. (2017). MDID: A multiply distorted image database for image quality assessment. Pattern Recognit..

[65] Bosc E., Pepion R., Le Callet P., Koppel M., Ndjiki-Nya P., Pressigout M., Morin L. (2011). Towards a new quality metric for 3-D synthesized view assessment. IEEE J. Sel. Top. Signal Process..

[66] Yang H., Fang Y., Lin W., Wang Z. (2014). Subjective quality assessment of screen content images. Proceedings of the 2014 Sixth International Workshop on Quality of Multimedia Experience (QoMEX).

[67] Ni Z., Ma L., Zeng H., Chen J., Cai C., Ma K.K. (2017). ESIM: Edge similarity for screen content image quality assessment. IEEE Trans. Image Process..

[68] Lin H., Hosu V., Saupe D. (2019). KADID-10k: A large-scale artificially distorted IQA database. Proceedings of the 2019 Eleventh International Conference on Quality of Multimedia Experience (QoMEX).

[69] Hosu V., Agnolucci L., Wiedemann O., Iso D., Saupe D. (2024). Uhd-iqa benchmark database: Pushing the boundaries of blind photo quality assessment. Proceedings of the European Conference on Computer Vision.

[70] Wang Z.J., Montoya E., Munechika D., Yang H., Hoover B., Chau D.H. (2023). Diffusiondb: A large-scale prompt gallery dataset for text-to-image generative models. Proceedings of the 61st Annual Meeting of the Association for Computational Linguistics (Volume 1: Long Papers).

[71] Wu X., Sun K., Zhu F., Zhao R., Li H. Human preference score: Better aligning text-to-image models with human preference. Proceedings of the IEEE/CVF International Conference on Computer Vision.

[72] Xu J., Liu X., Wu Y., Tong Y., Li Q., Ding M., Tang J., Dong Y. Imagereward: Learning and evaluating human preferences for text-to-image generation. Proceedings of the Advances in Neural Information Processing Systems (NeurIPS).

[73] Kirstain Y., Polyak A., Singer U., Matiana S., Penna J., Levy O. Pick-a-pic: An open dataset of user preferences for text-to-image generation. Proceedings of the Advances in Neural Information Processing Systems (NeurIPS).

[74] Yuan J., Li J., Yang F., Cao X., Che J., Lin J., Cao X. Pku-aigiqa-4k: A perceptual quality assessment database for both text-to-image and image-to-image ai-generated images. Proceedings of the IEEE/CVF International Conference on Computer Vision.

[75] Tian Y., Li Y., Chen B., Zhu H., Wang S., Kwong S. AI-generated image quality assessment in visual communication. Proceedings of the AAAI Conference on Artificial Intelligence.

[76] Li Y., Wu S., Sun W., Zhang Z., Zhu Y., Zhang Z., Duan H., Min X., Zhai G. (2026). AGHI-QA: A Subjective-Aligned Dataset and Metric for AI-Generated Human Images. IEEE Trans. Circuits Syst. Video Technol..

[77] Hernandez-Ortega J., Galbally J., Fierrez J., Haraksim R., Beslay L. Faceqnet: Quality assessment for face recognition based on deep learning. Proceedings of the 2019 International Conference on Biometrics (ICB).

[78] Terhorst P., Kolf J.N., Damer N., Kirchbuchner F., Kuijper A. SER-FIQ: Unsupervised Estimation of Face Image Quality Based on Stochastic Embedding Robustness. Proceedings of the IEEE/CVF Conference on Computer Vision and Pattern Recognition.

[79] Fang Y., Zhu H., Zeng Y., Ma K., Wang Z. Perceptual quality assessment of smartphone photography. Proceedings of the IEEE/CVF Conference on Computer Vision and Pattern Recognition.

[80] Chen C., Wei J., Peng C., Qin H. (2021). Depth-quality-aware salient object detection. IEEE Trans. Image Process..

[81] Ji W., Li J., Yu S., Zhang M., Piao Y., Yao S., Bi Q., Ma K., Zheng Y., Lu H. Calibrated RGB-D salient object detection. Proceedings of the IEEE/CVF Conference on Computer Vision and Pattern Recognition.

[82] Yeganeh H., Wang Z. (2012). Objective quality assessment of tone-mapped images. IEEE Trans. Image Process..

[83] Yang J., Zhou Y., Zhao Y., Wen J. (2022). Blind quality assessment of tone-mapped images using multi-exposure sequences. J. Vis. Commun. Image Represent..

[84] Liu J., Li X., Peng Y., Yu T., Chen Z. Swiniqa: Learned swin distance for compressed image quality assessment. Proceedings of the IEEE/CVF Conference on Computer Vision and Pattern Recognition.

[85] Yang M., Sowmya A. (2015). An underwater color image quality evaluation metric. IEEE Trans. Image Process..

[86] Xian W., Zhou M., Li Z. (2024). PIGUIQA: A physical imaging guided perceptual framework for underwater image quality assessment. arXiv.

[87] Zhang H., Li S., Li D., Wang Z., Zhou Q., You Q. (2022). Sonar image quality evaluation using deep neural network. IET Image Process..

[88] Chen W., Lin R., Zhang R., Zhu Y. (2022). A blind contour-aware quality model for sonar images. IET Image Process..

[89] Agnolucci L., Galteri L., Bertini M. (2024). Quality-Aware Image-Text Alignment for Real-World Image Quality Assessment. arXiv.

[90] Zhao Z., Yue X., Sun J., Xie Y., Shao T., Yao L., Xia F., Deng Y. iDETEX: Empowering MLLMs for Intelligent DETailed EXplainable IQA. Proceedings of the IEEE/CVF International Conference on Computer Vision.

[91] You Z., Cai X., Gu J., Xue T., Dong C. Teaching Large Language Models to Regress Accurate Image Quality Scores Using Score Distribution. Proceedings of the IEEE/CVF Conference on Computer Vision and Pattern Recognition (CVPR).

[92] You Z., Gu J., Li Z., Cai X., Zhu K., Dong C., Xue T. (2024). Descriptive image quality assessment in the wild. arXiv.

[93] Li W., Zhang X., Zhao S., Zhang Y., Li J., Zhang L., Zhang J. Q-Insight: Understanding Image Quality via Visual Reinforcement Learning. Proceedings of the Advances in Neural Information Processing Systems.

[94] Wu T., Zou J., Liang J., Zhang L., Ma K. (2025). Visualquality-r1: Reasoning-induced image quality assessment via reinforcement learning to rank. arXiv.

[95] Seshadrinathan K., Soundararajan R., Bovik A.C., Cormack L.K. (2010). Study of subjective and objective quality assessment of video. IEEE Trans. Image Process..

[96] Nuutinen M., Virtanen T., Vaahteranoksa M., Vuori T., Oittinen P., Häkkinen J. (2016). CVD2014—A Database for Evaluating No-Reference Video Quality Assessment Algorithms. IEEE Trans. Image Process..

[97] Ghadiyaram D., Pan J., Bovik A.C., Moorthy A.K., Panda P., Yang K.C. (2017). In-Capture Mobile Video Distortions: A Study of Subjective Behavior and Objective Algorithms. IEEE Trans. Circuits Syst. Video Technol..

[98] Hosu V., Hahn F., Jenadeleh M., Lin H., Men H., Szirányi T., Li S., Saupe D. The Konstanz natural video database (KoNViD-1k). Proceedings of the 2017 Ninth International Conference on Quality of Multimedia Experience (QoMEX).

[99] Sinno Z., Bovik A.C. (2018). Large-scale study of perceptual video quality. IEEE Trans. Image Process..

[100] Wang Y., Inguva S., Adsumilli B. (2019). YouTube UGC dataset for video compression research. Proceedings of the 2019 IEEE 21st International Workshop on Multimedia Signal Processing (MMSP).

[101] Ying Z., Mandal M., Ghadiyaram D., Bovik A. (2021). Patch-VQ: ‘Patching Up’ the Video Quality Problem. arXiv.

[102] Bampis C.G., Li Z., Katsavounidis I., Huang T.Y., Ekanadham C., Bovik A.C. (2021). Towards perceptually optimized adaptive video streaming-a realistic quality of experience database. IEEE Trans. Image Process..

[103] Duanmu Z., Liu W., Li Z., Chen D., Wang Z., Wang Y., Gao W. (2020). The Waterloo Streaming Quality-of-Experience Database-IV. IEEE Dataport. http://ieee-dataport.org/open-access/waterloo-streaming-quality-experience-database-iv.

[104] Duan H., Hu Q., Wang J., Yang L., Xu Z., Liu L., Min X., Cai C., Ye T., Zhang X. FineVQ: Fine-Grained User Generated Content Video Quality Assessment. Proceedings of the IEEE/CVF Conference on Computer Vision and Pattern Recognition.

[105] Jindal A., Sadaka N., Thomas M.M., Sochenov A., Kaplanyan A. (2025). CGVQM+D: Computer Graphics Video Quality Metric and Dataset. Comput. Graph. Forum.

[106] Safonov N., Rakhmanov M., Vatolin D., Timofte R., Wu C., Wu K., Patro K., Rathour P., Channappayya S., Pardhi P. AIM 2025 Challenge on Screen-Content Video Quality Assessment: Methods and Results. Proceedings of the IEEE/CVF International Conference on Computer Vision (ICCV) Workshops.

[107] Pu Y., Li K., Huang Z., Zhong Z., Yang K. (2025). MVQA-68K: A Multi-Dimensional and Causally-Annotated Dataset with Quality Interpretability for Video Assessment. Proceedings of the 33rd ACM International Conference on Multimedia.

[108] Chivileva I., Lynch P., Ward T.E., Smeaton A.F. (2023). Measuring the Quality of Text-to-Video Model Outputs: Metrics and Dataset. arXiv.

[109] Feng W., Li J., Saxon M., Fu T.j., Chen W., Wang W.Y. (2024). Tc-bench: Benchmarking temporal compositionality in text-to-video and image-to-video generation. arXiv.

[110] Liao M., Lu H., Zhang X., Wan F., Wang T., Zhao Y., Zuo W., Ye Q., Wang J. (2024). Evaluation of text-to-video generation models: A dynamics perspective. Adv. Neural Inf. Process. Syst..

[111] Sun K., Huang K., Liu X., Wu Y., Xu Z., Li Z., Liu X. T2v-compbench: A comprehensive benchmark for compositional text-to-video generation. Proceedings of the Computer Vision and Pattern Recognition Conference.

[112] Vu P.V., Chandler D.M. (2014). ViS3: An algorithm for video quality assessment via analysis of spatial and spatiotemporal slices. J. Electron. Imaging.

[113] Soundararajan R., Bovik A.C. (2013). Video Quality Assessment by Reduced Reference Spatio-Temporal Entropic Differencing. IEEE Trans. Circuits Syst. Video Technol..

[114] Mittal A., Saad M.A., Bovik A.C. (2015). A completely blind video integrity oracle. IEEE Trans. Image Process..

[115] Kancharla P., Channappayya S.S. (2022). Completely Blind Quality Assessment of User Generated Video Content. IEEE Trans. Image Process..

[116] Tu Z., Yu X., Wang Y., Birkbeck N., Adsumilli B., Bovik A.C. (2021). RAPIQUE: Rapid and accurate video quality prediction of user generated content. IEEE Open J. Signal Process..

[117] Wu H., Chen C., Liao L., Hou J., Sun W., Yan Q., Lin W. (2023). DisCoVQA: Temporal Distortion-Content Transformers for Video Quality Assessment. IEEE Trans. Circuits Syst. Video Technol..

[118] Wei H., Chen C., Liao L., Hou J., Wu H., Sun W., Yan Q., Lin W. (2023). Learning Spatiotemporal Interactions for User-Generated Video Quality Assessment. IEEE Trans. Circuits Syst. Video Technol..

[119] Liu Y., Quan Y., Xiao G., Li A., Wu J. Scaling and masking: A new paradigm of data sampling for image and video quality assessment. Proceedings of the AAAI Conference on Artificial Intelligence.

[120] Wu H., Zhang E., Liao L., Chen C., Hou J.H., Wang A., Sun W.S., Yan Q., Lin W. Exploring Video Quality Assessment on User Generated Contents from Aesthetic and Technical Perspectives. Proceedings of the International Conference on Computer Vision (ICCV).

[121] Wen W., Li M., Zhang Y., Liao Y., Li J., Zhang L., Ma K. Modular Blind Video Quality Assessment. Proceedings of the IEEE/CVF Conference on Computer Vision and Pattern Recognition.

[122] Cao L., Sun W., Zhang W., Zhu X., Jia J., Zhang K., Zhu D., Zhai G., Min X. (2025). VQAThinker: Exploring Generalizable and Explainable Video Quality Assessment via Reinforcement Learning. arXiv.

[123] Jia Z., Zhang Z., Qian J., Wu H., Sun W., Li C., Liu X., Lin W., Zhai G., Min X. (2025). VQA2: Visual Question Answering for Video Quality Assessment. Proceedings of the 33rd ACM International Conference on Multimedia.

[124] Wu S., Li Y., Xu Z., Gao Y., Duan H., Sun W., Zhai G. (2025). FVQ: A Large-Scale Dataset and an LMM-Based Method for Face Video Quality Assessment. Proceedings of the 33rd ACM International Conference on Multimedia.

[125] Fang Y., Li Z., Yan J., Sui X., Liu H. (2023). Study of spatio-temporal modeling in video quality assessment. IEEE Trans. Image Process..

[126] Wang X., Katsenou A., Shen J., Bull D. CAMP-VQA: Caption-Embedded Multimodal Perception for No-Reference Quality Assessment of Compressed Video. Proceedings of the IEEE/CVF Winter Conference on Applications of Computer Vision.

[127] Zhang X., Li W., Zhao S., Li J., Zhang L., Zhang J. Vq-insight: Teaching vlms for ai-generated video quality understanding via progressive visual reinforcement learning. Proceedings of the AAAI Conference on Artificial Intelligence.

[128] Sun M., Kong G., Duan X., Long H. (2025). Memory-VQA: Video quality assessment of UGC based on human memory system. Neurocomputing.

[129] Wu H., Zhang Z., Zhang W., Chen C., Liao L., Li C., Gao Y., Wang A., Zhang E., Sun W. (2023). Q-align: Teaching lmms for visual scoring via discrete text-defined levels. arXiv.

[130] Alexiou E., Ebrahimi T. (2017). On the performance of metrics to predict quality in point cloud representations. Proceedings of the Applications of Digital Image Processing XL.

[131] Alexiou E., Upenik E., Ebrahimi T. (2017). Towards subjective quality assessment of point cloud imaging in augmented reality. Proceedings of the 2017 IEEE 19th International Workshop on Multimedia Signal Processing (MMSP).

[132] Alexiou E., Ebrahimi T., Bernardo M.V., Pereira M., Pinheiro A., Cruz L.A.D.S., Duarte C., Dmitrovic L.G., Dumic E., Matkovics D. (2018). Point cloud subjective evaluation methodology based on 2D rendering. Proceedings of the 2018 Tenth International Conference on Quality of Multimedia Experience (QoMEX).

[133] Zerman E., Gao P., Ozcinar C., Smolic A. (2019). Subjective and objective quality assessment for volumetric video compression. Soc. Imaging Sci. Technol..

[134] Javaheri A., Brites C., Pereira F., Ascenso J. (2020). Point cloud rendering after coding: Impacts on subjective and objective quality. IEEE Trans. Multimed..

[135] Liu Q., Su H., Duanmu Z., Liu W., Wang Z. (2023). Perceptual quality assessment of colored 3D point clouds. IEEE Trans. Vis. Comput. Graph..

[136] Liu Q., Yuan H., Hamzaoui R., Su H., Hou J., Yang H. (2021). Reduced reference perceptual quality model with application to rate control for video-based point cloud compression. IEEE Trans. Image Process..

[137] Ak A., Zerman E., Quach M., Chetouani A., Smolic A., Valenzise G., Le Callet P. (2024). BASICS: Broad Quality Assessment of Static Point Clouds in a Compression Scenario. IEEE Trans. Multimed..

[138] Nehmé Y., Dupont F., Farrugia J.P., Le Callet P., Lavoué G. (2020). Visual quality of 3d meshes with diffuse colors in virtual reality: Subjective and objective evaluation. IEEE Trans. Vis. Comput. Graph..

[139] Nehmé Y., Delanoy J., Dupont F., Farrugia J.P., Le Callet P., Lavoué G. (2023). Textured mesh quality assessment: Large-scale dataset and deep learning-based quality metric. ACM Trans. Graph..

[140] Zhang Z., Zhou Y., Sun W., Lu W., Min X., Wang Y., Zhai G. (2023). Ddh-qa: A dynamic digital humans quality assessment database. Proceedings of the 2023 IEEE International Conference on Multimedia and Expo (ICME).

[141] Liu Y., Zhang Y., Yang Q., Xu Y., Li Z., Wang Y.K. (2025). DPCD: A Quality Assessment Database for Dynamic Point Clouds. Proceedings of the 2025 IEEE International Conference on Multimedia and Expo (ICME).

[142] Li Y., Wu S., Zhu Y., Duan H., Zhang Z., Zhai G. (2025). DHQA-4D: Perceptual Quality Assessment of Dynamic 4D Digital Human. arXiv.

[143] Zhang Y., Yang Q., Xu Y. (2021). MS-GraphSIM: Inferring Point Cloud Quality via Multiscale Graph Similarity. Proceedings of the 29th ACM International Conference on Multimedia.

[144] Javaheri A., Brites C., Ascenso J. A Point-to-Distribution Joint Geometry and Color Metric for Point Cloud Quality Assessment. Proceedings of the IEEE International Workshop on Multimedia Signal Processing (MMSP).

[145] Lu W., Gu S., Zhang Y., Xu Y. Machine Perception Point Cloud Quality Assessment via Vision Tasks. Proceedings of the International Conference on 3D Immersion (IC3D).

[146] Yang Q., Liu Y., Chen S., Xu Y., Sun J. No-Reference Point Cloud Quality Assessment via Domain Adaptation. Proceedings of the IEEE/CVF Conference on Computer Vision and Pattern Recognition (CVPR).

[147] Zhang Z., Sun W., Wu H., Zhou Y., Li C., Chen Z., Min X., Zhai G., Lin W. (2024). Gms-3dqa: Projection-based grid mini-patch sampling for 3d model quality assessment. ACM Trans. Multimed. Comput. Commun. Appl..

[148] Zhang Z., Sun W., Zhu Y., Min X., Wu W., Chen Y., Zhai G. (2023). Evaluating Point Cloud from Moving Camera Videos: A No-Reference Metric. IEEE Trans. Multimed..

[149] Shan Z., Zhang Y., Yang Q., Yang H., Xu Y., Hwang J.N., Xu X., Liu S. Contrastive pre-training with multi-view fusion for no-reference point cloud quality assessment. Proceedings of the IEEE/CVF Conference on Computer Vision and Pattern Recognition.

[150] Yang Q., Xu Y., Tang R., Peng Z., Ma Z., Yang K. (2025). MPV-PCQA: Multimodal No-Reference Point Cloud Quality Assessment via Point Cloud and Captured Dynamic Video. Multimed. Syst..

[151] (2025). BMPCQA: Bioinspired Metaverse Point Cloud Quality Assessment Based on Large Multimodal Models. Adv. Intell. Syst..

[152] Xu R., Wang X., Wang T., Chen Y., Pang J., Lin D. (2024). PointLLM: Empowering Large Language Models to Understand Point Clouds. Proceedings of the European Conference on Computer Vision (ECCV).

